# Impact of diabetes mellitus on the menopausal experience: A scoping review

**DOI:** 10.1111/dme.70420

**Published:** 2026-08-22

**Authors:** Rachel Black, Felicity Hasson, Lisa King, Mark Davies, Deborah Muldrew, Paul Slater, Jenny Johnston, Dawn Adams, Rhonda Murphy, George Kernohan, Vivien Coates

**Affiliations:** ^1^ Institute of Nursing and Health Research Ulster University Belfast UK; ^2^ Institute of Nursing and Health Research, School of Nursing & Paramedic Sciences Ulster University Belfast UK; ^3^ Institute of Nursing and Health Research, School of Nursing and Paramedic Science Ulster University Belfast Campus Belfast UK; ^4^ Clinical Psychology Department Belfast City Hospital Belfast UK; ^5^ Statistician Institute of Nursing and Health Research Ulster University Belfast UK; ^6^ Centre for Medical Education Queen's University Belfast, Whitla Medical Building Belfast UK; ^7^ Midwifery Southern Health and Social Care Trust Portadown UK; ^8^ Derry Well Woman Derry UK; ^9^ Institute of Nursing and Health Research, School of Nursing and Paramedic Science Ulster University, Derry‐Londonderry Campus Londonderry UK

**Keywords:** diabetes, menopause, peri‐menopause, scoping review

## Abstract

**Aims:**

To identify, appraise and synthesise the current evidence relating to symptoms, experiences, management and impact of menopause on QoL among women with type 1 diabetes (T1DM) or type 2 diabetes (T2DM) and to identify evidence gaps.

**Methods:**

We conducted a scoping review guided by Arksey and O'Mally's framework by searching CINAHL, MEDLINE, EMBASE, PsychINFO and PubMed using an iterative approach to pre‐specified eligibility criteria which were reviewed with increasing familiarity with the literature. Studies focusing on peri‐menopausal and menopausal people assigned female at birth with a diagnosis of T1DM or T2DM were included.

**Results:**

In total, 16,681 records were identified from which 77 papers were subjected to full review, with 22 eligible. Research focus and methodology varied: 16 observational studies, 2 expert consensus reviews, 2 interventional studies, 1 patient and public involvement paper and 1 qualitative grounded theory paper. PRISMA‐ScR guidelines were followed for transparency of reporting. Findings indicate women with diabetes experience menopausal symptoms similar to those of women without diabetes; however, they may be at risk of increased severity of symptoms, HRT use was linked to lower HbA1c levels but is largely overlooked, limited research has been undertaken exploring women's experiences and awareness of support during the menopause; existing research evidence suggests they are ill‐informed and lacking effective evidence based support. There is a particular dearth of evidence relating to women with T1DM.

**Conclusions:**

This review provides evidence that menopause has a negative influence upon quality of life for many women with diabetes. The impacts span managing diabetes and menopausal symptoms and seeking and receiving support. A hypothesised bidirectional relationship between diabetes and menopause may exist: whereby progression of one may be influenced by the other also resulting in a lower quality of life. While high‐level evidence on this complex inter‐relationship remains limited, particularly in T1DM populations, it suggests significant unmet need for those experiencing diabetes and menopause together.

**What's new?**

- The review confirms that the menopause transition has a negative impact on women with diabetes with a suggested increased risk of greater psychological distress, increased menopause symptom severity and negative impact on QoL for women living with T2DM. Furthermore, it is tentatively hypothesised that there is a bidirectional interplay between diabetes and menopause.
- Qualitative evidence suggests women are largely uninformed about menopause with limited guidance on managing both diabetes and menopause, while their concerns are often dismissed by their care teams. Women with T1DM report confusion of vasomotor symptoms with hypoglycaemic episodes highlighting a T1DM specific risk for effective diabetes management.
- Most studies focus on T2DM, highlighting a significant gap in current literature which includes the unique experiences and needs of women with T1DM during the menopause. More research is needed to explore the impact of menopause on QoL in this population.

## 1 INTRODUCTION

Menopause is a significant life event for women and people who menstruate, signifying the end of reproductive life. Peri‐menopause is the transitional period leading up to menopause, marked by fluctuating hormone levels, and can last up to around 8 years. Changes in endocrinological, biological and clinical features can be observed during this time. Natural menopause itself is defined clinically as being 1 year since the last menstrual period. Surgical menopause (removal of the ovaries), early menopause (<45 years) and premature ovarian insufficiency (<40 years and often autoimmune) are also seen. While the trajectory towards menopause varies, the peri‐menopausal stage typically begins between the ages of 45 and 55 years.^1^ In May 2025, it was estimated that more than one billion women worldwide were experiencing peri‐menopause, menopause or were post‐menopausal. By 2030, an estimated 500 million women will reach the age of 45–55 years, suggesting that 6% of the global population will be in menopause.

Both peri‐menopause and menopause are associated with an array of physical and psychological symptoms. In addition to the well‐known vasomotor changes, effects can be seen on nervous system‐related disorders; metabolic, weight, cardiovascular, musculoskeletal changes; urogenital and skin atrophy; and sexual dysfunction.^2^, ^3^ While symptoms can significantly vary, women with increased severity of symptoms are at risk of lower quality of life (QoL).^4^, ^5^ Women diagnosed with diabetes may face greater challenges during peri‐menopause and menopause due to an interplay of symptoms from both conditions caused by hormonal fluctuations and the impact on insulin sensitivity.^6^

Previous research indicates that peri‐menopause exerts common effects in relation to diabetes and metabolic function.^2^, ^7^ Weight gain, impaired insulin secretion and sensitivity, impaired glucose tolerance (IGT), hypertension and hypercholesterolaemia have been found to be affected by hormonal changes during menopause.^8^, ^9^ In addition, the physical and emotional symptoms related to both diabetes and menopause can include vasomotor symptoms (hot‐flushes, night‐sweats; sleep disturbances; mood swings, increased risk of depressive symptoms; and sexual dysfunction).^10^, ^11^

Despite the above studies and rising incidence of diabetes diagnosis among women^12^ the impact of menopause for women living with diabetes is relatively unknown.^13^ These conditions are recognised to be challenging and often require medical and emotional support. The needs of those experiencing both are not fully understood.

A recent Diabetes UK position statement identifies several areas for future research focus.^14^ Recommendations specified: (1) improved understanding of the mechanisms underlying the impact of menopause on blood glucose control in women with diabetes; (2) Understanding the safety and efficacy of treatments, technology and exercise in the management of diabetes and menopause; (3) Understanding how to best support women from underserved communities in their management of diabetes and menopause; (4) Understanding the impact of menopause on the mental health of women with diabetes and tailoring psychosocial interventions that support women during this period; (6) Supporting healthcare professional education in how to effectively support women with diabetes experiencing menopause; and (7) exploring how innovative models of care can support women with diabetes experiencing menopause more effectively.

The current lack of understanding and professional expertise is a pertinent consideration for ensuring women's well‐being during this transitional stage. The concept of QoL aims to incorporate broad concepts such as ‘*personal health (physical, mental, and spiritual), relationships, education status, work environment, social status, wealth, a sense of security and safety, freedom, autonomy in decision‐making, social belonging, and physical surroundings*’.^15^ While health‐related quality of life (HRQoL) specifically explores the links between health and QoL. Individuals living with diabetes have been found to experience an overall lower general QoL and HRQoL^16^ with studies demonstrating being female as a factor contributing to diminished QoL.^17^, ^18^ Additionally, research exploring QoL during menopause has found that peri‐menopause and ‐menopause elicits a trend towards lower QoL in women experiencing more severe menopause symptoms.^5^

Therefore, a better understanding of the complexities and impact of living with diabetes during this significant life stage has on women's QoL is required. There are no reviews known to the authors that systematically summarise the current evidence base for both type 1 diabetes (T1DM) and type 2 diabetes (T2DM). Accordingly, a scoping review to identify the breadth of evidence and highlight gaps was undertaken.

## 2 AIMS

To identify, appraise and synthesise the current evidence relating to symptoms, experiences, management and impact of menopause on QoL among women with T1DM or T2DM and to identify evidence gaps. This review focuses on the primary research question ‘What is known from the literature about women's experiences of living with diabetes and menopause and its impact on their quality of life?’

The following subquestions were explored:

1. How do women with T1DM or T2DM experience the symptoms of menopause?
2. What are women's experiences of managing diabetes during menopause?
3. What help seeking behaviours do women with T1DM or T2DM adopt during menopause?
4. What is the impact of menopause on QoL in women with diabetes?

## 3 METHODS

This scoping review followed the five‐stage methodological framework outlined by Arksey and O'Malley.^19^ Furthermore, the optional 6th step ‘consultation exercise’ was incorporated throughout the review process. Stakeholders including clinicians, experts by experience and other voluntary organisations were consulted to inform the focus of the review and validate findings. Recommendations from Levac et al.^20^ and the Joanna Briggs Institute^21^ (JBI) were adopted to enhance clarity in reporting. A review protocol was developed and is available upon request from the corresponding author.

The reporting of results followed the PRISMA Extension for Scoping Reviews (PRISMA‐ScR)^22^ (See File S1).

### 3.1 Search strategy

Five electronic databases (CINAHL, MEDLINE, EMBASE, PsychINFO and PubMed) were systematically searched, including backward and forward citation tracking from the included articles, using pre‐specified eligibility criteria in June 2024. This was supplemented by grey literature searches to ensure a comprehensive approach was adopted.

The Population, Concept, Context (PCC) mnemonic recommended by JBI^21^ was used to structure the development of key terms, aligned to the objectives of the review.

- *Population*: Women with T1DM/T2DM with experience of peri/menopausal symptoms
- *Concept*: Living with T1DM or T2DM through menopause
- *Context*: Globally

Key terms were developed with input from key stakeholders, experts by experience and a subject specialist librarian. Example combinations of keywords used include: ‘Diabetes’, ‘Type 1 Diabetes’, ‘Type 2 Diabetes’, menopause (menopaus*), peri‐menopause (peri‐menopaus*). Searches for articles were conducted using keywords and Boolean terms AND/OR alongside a combination of the appropriate Medical Subject Headings terms. The results were cross‐checked to ensure seminal academic work in the area was captured in the results. Full search can be found in File S2.

### 3.2 Study selection

Eligibility was iteratively developed according to increasing familiarity with the literature, with some minor modifications to the original review protocol to include papers reporting on surgical menopause, and a broad approach when operationalising the inclusion term of ‘experience’ was agreed by the research team. The inclusion criteria were:

1. Papers published in English Language, full text, utilising any research design (qualitative, quantitative or mixed‐method methodologies), empirical review, discussion and editorial papers which report empirical data, grey literature. No limits were set on country of origin.
2. Studies focusing on peri‐menopausal and menopausal people assigned female at birth with a diagnosis of T1DM or T2DM were included. Studies including peri/menopausal women or both based on clinical diagnosis or self report were included.

#### 3.2.1 Exclusion criteria

Articles were excluded if they reported on:

1. Studies with no explicit menopausal status reported.
2. Studies mixing samples with and without diabetes were not considered, unless separate analyses for individuals with diabetes and without diabetes were presented.
3. Reviews and studies without empirical data such as opinion pieces.
4. Studies in which women only developed diabetes when post‐menopause.

### 3.3 Data extraction

Search results were compiled by one member of the research team (RB) and merged using reference management software before duplicates were identified and removed. Five independent investigators screened titles and abstracts against eligibility criteria, followed by full text screening of the remaining papers. Disagreement at each stage of the selection process was resolved through discussion and consensus.

A data charting form was developed (based on the JBI extraction instrument template) to address the study questions. Relevant information procured from the literature was extracted and tabulated using this format. The charting form was used to record basic citation information, data about PCC, methodology of the studies, main findings, summary of the results, strengths and limitations of the studies, and research gaps proposed by the authors. Adhering to methodological guidance^19^, ^20^, ^21^ quality assessment was not undertaken. This is in keeping with scoping methodology, given the heterogeneity of papers involved. The synthesis and reporting of evidence within this review was guided by a hierarchical weighting of study designs, adapted to the scoping review framework which prioritises the mapping of heterogeneity over statistical pooling.^23^

## 4 RESULTS

Searches were conducted in June 2024. A total of 16,681 records were identified through the database searches. After duplicates were removed 9887 titles and abstracts were screened leaving 77 full‐text articles to be assessed for eligibility. A total of 22 studies were included^6^, ^9^, ^10^, ^11^, ^24^, ^25^, ^26^, ^27^, ^28^, ^29^, ^30^, ^31^, ^32^, ^33^, ^34^, ^35^, ^36^, ^37^, ^38^, ^39^, ^40^, ^41^ The results of the study inclusion process are presented in a PRISMA‐ScR flow diagram for transparency (Figure 1).

**FIGURE 1:**
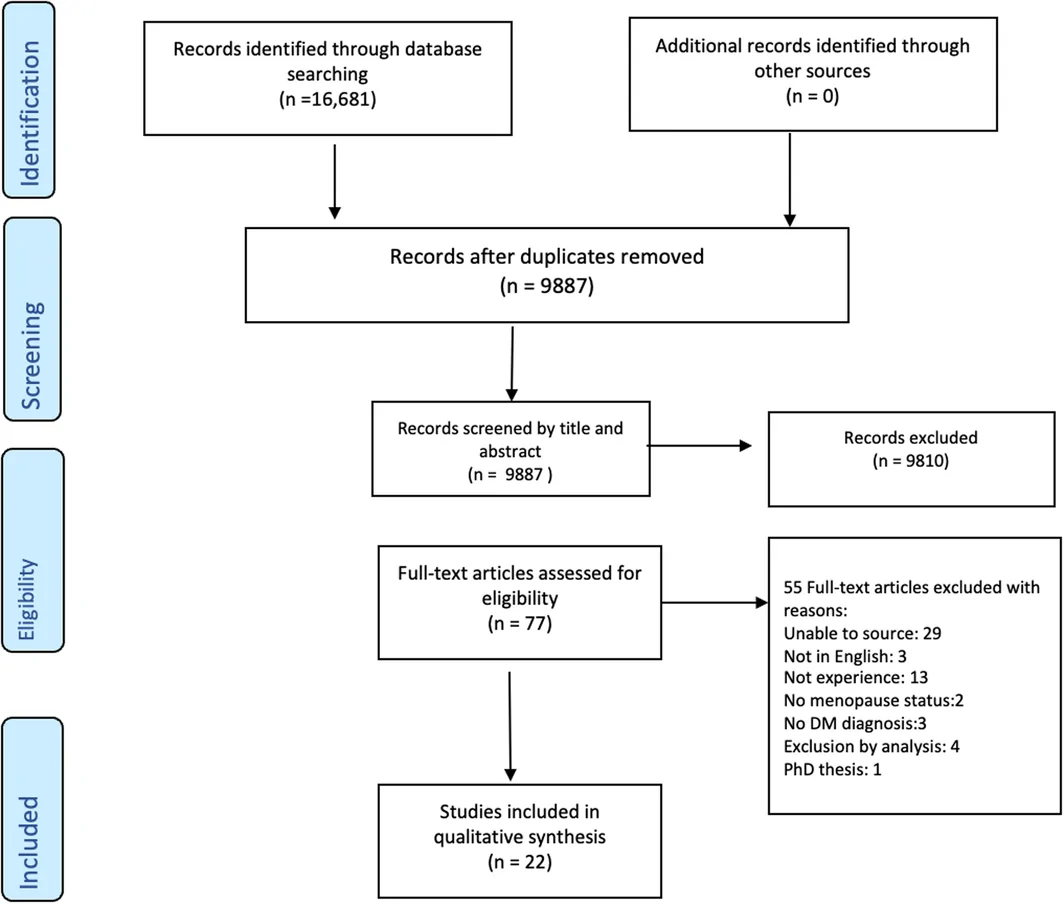
PRISMA‐ScR flow diagram for study selection.

### 4.1 Study characteristics

The included papers represented a wide geographical spread: United States^24^, ^31^, ^34^, ^39^; United Kingdom^6^, ^26^, ^36^, ^41^; Mexico^25^, ^27^, ^37^; Poland^33^, ^35^; Greece^9^, ^40^; Malaysia^29^, ^30^; Australia^36^; India^38^; China^34^; Iran^32^; Brazil^11^; Peru^10^ from 17,531 women living with diabetes (T1DM *n* = 194; T2DM *n* = 17,337). There were no studies from Africa.

The studies were varied in their research focus and methodology with 16 observational studies,^10^, ^11^, ^24^, ^25^, ^26^, ^27^, ^28^, ^31^, ^33^, ^34^, ^35^, ^37^, ^38^, ^39^ 2 expert consensus reviews,^9^, ^40^ 2 interventional studies,^32^, ^36^ 1 patient and public involvement paper (PPI)^41^ and 1 qualitative grounded theory.^6^ Most of the included studies focused on T2DM (*n* = 16).^9^, ^10^, ^11^, ^24^, ^25^, ^27^, ^28^, ^29^, ^30^, ^31^, ^32^, ^34^, ^35^, ^36^, ^37^, ^39^ Three papers explored T1DM,^6^, ^26^, ^41^ one examined T1DM and T2DM^40^ and two papers did not specify the type of diabetes.^33^, ^38^ Fifteen papers presented empirical research with a primary focus on the interaction between diabetes and menopause by exploring factors such as psychological and physiological symptoms of menopause, metabolic characteristics, and help‐seeking and management.^6^, ^10^, ^11^, ^24^, ^25^, ^26^, ^27^, ^28^, ^29^, ^30^, ^31^, ^32^, ^33^, ^35^, ^38^ Publication dates ranged from 1997 to 2024. A summary of all papers including primary and secondary outcomes and funding source can be found in File S3.

### 4.2 Menopause symptom mapping

Whilst not all papers focused on menopause symptoms in women living with diabetes as their primary aim^29^, ^30^, ^31^, ^33^, ^35^, ^36^ those who reported any menopause‐related symptoms within papers were mapped. The findings demonstrate that menopausal women with T1DM and T2DM experience a plethora of symptoms ranging from psychological, vasomotor, genitourinary and physical issues. There was a paucity of research focusing solely on menopausal symptom prevalence and severity in women living with diabetes. However, the lack of a standard methodological approach to outcome measurement made it impossible to calculate symptom prevalence within this population.

#### 4.2.1 Genitourinary symptoms of menopause

Genitourinary symptoms reported within the included studies are presented in Table 1.

**TABLE 1:** Genitourinary symptom mapping across studies.

Symptom	Source	Menopause status	Diabetes type
Vaginal dryness	Mackay et al.^6^	Peri/menopausal	T1DM
	Hasan et al.^30^	Post‐menopausal	T2DM
	Parsa et al.^32^	Menopausal	T2DM
Urinary incontinence	Mackay et al.^6^	Peri/menopausal	T1DM
	Rouen et al.^31^	Post‐menopausal	T2DM
Vaginal irritation	Rouen et al.^31^	Post‐menopausal	T2DM
Urogenital symptoms (unspecified)	Zimny et al.^35^	Peri‐menopausal	T2DM
Pain during intercourse	Hasan et al.^30^ (post‐menopausal)	Post‐menopausal	T2DM
	Inuy et al.^11^ (post‐menopausal, 23%)	Post‐menopausal	T2DM
	Mezones‐Holguin et al.^10^	Post‐menopausal	T2DM
Orgasmic dysfunction	Inuy et al.^11^	Post‐menopausal	T2DM
	Mezones‐Holguin et al.^10^	Post‐menopausal	T2DM
Reduced desire	Inuy et al.^11^	Post‐menopausal	T2DM
	Mezones‐Holguin et al.^10^	Post‐menopausal	T2DM
Reduced satisfaction	Inuy et al.^11^	Post‐menopausal	T2DM
	Mezones‐Holguin et al.^10^	Post‐menopausal	T2DM
Sexual problems (unspecified)	Mackay et al.^6^	Peri/menopause	T1DM
Overall sexual dysfunction	Inuy et al.^11^	Post‐menopausal	T2DM
	Mezones‐Holguin et al.^10^	Post‐menopausal	T2DM
	Fang et al.^28^	Post‐menopausal	T2DM

Abbreviations: T1DM, type 1 diabetes mellitus; T2DM, type 2 diabetes mellitus.

Sexual symptoms commonly reported across the studies include loss of libido, vaginal dryness and satisfaction.

Three empirical studies explored female sexual dysfunction (FSD) in women with diabetes as the primary aim.^10^, ^11^, ^28^ They included women from Peruvian, Brazilian and Chinese ethnicities. All three studies focused on T2DM only.

Overall, the included papers suggest a higher FSD burden on women with diabetes with lower scores on the FSFI compared to controls (18–19 vs. 23–27, respectively) and higher overall FSD rates (75–85% vs. 31–55%). While Fang et al. reported overall significance, their sample included pre‐menopausal and post‐menopausal women. In subgroup analyses they report an insignificant difference in FSD prevalence between post‐menopausal women with diabetes (91%) compared with those without (84.4%, *p* > 0.05). This lack of significance could, however, be due to the small post‐menopausal sample and high control group FSD scores potentially indicating menopause itself is a major contributor and masking T2DM's additional effect in this subgroup.

Across the studies exploring FSD, age, menopause status and duration were noted to be risk factors alongside diabetes. Depression was found to amplify effects; however, diabetes was found to be an independent risk factor. Higher body mass^28^ and poor glycaemic control^10^ worsen outcomes in women with T2DM. However, there is no consensus on the link between diabetes duration and FSD.

The typology of evidence (Table 2) examining genitourinary symptoms in menopausal women with diabetes is dominated by Level 3 empirical cross‐sectional studies, which consistently demonstrate a higher burden of FSD in women with T2DM compared with non‐diabetic controls. These studies—conducted across diverse populations in Brazil, China and Peru—show clear deficits in lubrication, arousal, satisfaction and pain, indicating that diabetes amplifies key components of genitourinary syndrome of menopause (GSM). Level 4 methodological work confirms that sexual and urogenital domains meaningfully contribute to reduced QoL in women with T2DM, while Level 5 expert consensus proposes plausible biological mechanisms—such as hyperglycaemia‐related vascular and autonomic dysfunction—that may intensify GSM in this population. Collectively, the evidence base is moderate in strength but highly consistent in direction, indicating that genitourinary symptoms are more severe and more functionally disruptive in women with diabetes, particularly T2DM, even though causal pathways cannot be fully established due to the predominance of cross‐sectional designs.

**TABLE 2:** Typology of evidence—Genitourinary symptoms of menopause.

Author (year)	Categorisation	CEBM level^a^	Diabetes type	Population/Context	Key findings (genitourinary symptoms)
Inuy et al.^11^	Empirical—Quantitative (cross‐sectional)	Level 3	T2DM	184 post‐menopausal Brazilian women (100 with T2DM)	Higher FSD prevalence in T2DM (83% vs. 63%); lower FSFI scores, especially in lubrication, arousal and satisfaction domains
Fang et al.^28^	Empirical—Quantitative (cross‐sectional)	Level 3	T2DM	222 Chinese women (115 with T2DM)	Lower total FSFI scores in T2DM; worse lubrication and pain. Overall FSD higher in T2DM, but post‐menopausal subgroup difference not significant (91% vs. 84.4%)
Mezones‐Holguin et al.^10^	Empirical—Quantitative (cross‐sectional)	Level 3	T2DM	165 post‐menopausal women in Peru	Higher FSD risk in diabetic women; depression a stronger predictor than diabetes, but diabetes still an independent risk factor
Zimny et al.^35^	Empirical—Quantitative (cross‐sectional)	Level 3	T2DM	243 peri‐menopausal women (116 with T2DM)	Reported urogenital symptoms (unspecified) as part of broader QoL impairment in T2DM
Hasan et al.^30^	Methodological (validation study)	Level 4	T2DM	215 post‐menopausal women with T2DM (Malaysia)	MENQOL sexual domain (including vaginal dryness, pain with intercourse) contributed to overall QoL burden
Lambrinoudaki et al.^40^	Expert consensus/Narrative review	Level 5	T1DM/T2DM	Global clinical literature synthesis	Proposes that hyperglycaemia, inflammation and autonomic neuropathy impair vaginal blood flow and lubrication, potentially worsening GSM in women with diabetes

Abbreviations: T1DM, type 1 diabetes mellitus; T2DM, type 2 diabetes mellitus.

^a^ Levels range from 1 (highest level of evidence) to 5 (lowest level of evidence).

#### 4.2.2 Psychological symptoms

Psychological symptoms included depression, anxiety, mood swings, poor memory and dissatisfaction with personal life. Other psychological symptoms which were considered related to the impact of menopause included diabetes distress. Table 3 maps psychological symptoms across papers.

**TABLE 3:** Psychological symptom mapping across studies.

Symptom	Source	Menopause status	Diabetes type
Depression	Hasan et al.^29^	Post‐menopausal	T2DM
	Malacara et al.^25^	Post‐menopausal	T2DM
	Mezones‐Holguin et al.^10^	Post‐menopausal	T2DM
	Zimny et al.^35^	Peri‐menopausal	T2DM
	Florez et al.^37^	Menopausal	T2DM
Anxiety	Mackay et al.^6^	Peri/menopausal	T1DM
	Hasan et al.^29^	Post‐menopausal	T2DM
	Rouen et al.^31^	Post‐menopausal	T2DM
	Malacara et al.^25^	Post‐menopausal	T2DM
	Mezones‐Holguin et al.^10^	Post‐menopausal	T2DM
	Parsa et al.^32^	Menopausal	T2DM
Mood swings	Mackay et al.^6^	Peri/menopausal	T1DM
	Rouen et al.^31^	Post‐menopausal	T2DM
	Szadowska‐Szlachetka et al.^33^	Menopausal	T2DM
Poor memory	Hasan et al.^30^	Post‐menopausal	T2DM
	Tirupathi et al.^38^	Post‐menopausal	T2DM
Dissatisfaction with personal life	Hasan et al.^30^	Post‐menopausal	T2DM
	Tirupathi et al.^38^	Post‐menopausal	T2DM
	Szadowska‐Szlachetka et al.^33^	Menopausal	T2DM
Diabetes distress	Anderson et al.^36^	Peri‐/post‐menopausal	T2DM
Perceptions of aging/mortality	Mackay et al.^6^	Peri/menopausal	T1DM
	Szadowska‐Szlachetka et al.^33^	Menopausal	T2DM
Fear (e.g., hypoglycaemia)	Mackay et al.^6^	Peri/menopausal	T1DM
Irritability	Parsa et al.^32^	Menopausal	T2DM
Empty nest syndrome	Malacara et al.^25^	Post‐menopausal	T2DM

Abbreviations: T1DM, type 1 diabetes mellitus; T2DM, type 2 diabetes mellitus.

Findings revealed anxiety and depression as the most commonly cited psychological symptoms reported in the included papers. Depression scores were explicitly reported in four papers comparing women living with T2DM diabetes with a non‐diabetes control group; two reported significant differences in depression scores, with women who have T2DM scoring higher than the control group.^10^, ^25^ This inconsistency in significance of findings between the four papers likely reflects differences in sample characteristics, measurement tools and cultural context. Differences in the number of participants reporting anxiety and depression according to ethnicity were noted in one paper. Anxiety was more commonly reported by Chinese women, while depression was more frequently found in those of Indian ethnicity.^29^

A pertinent observation in relation to diabetes management was noted by Florez et al.^37^ who explored medication use and psychosocial factors including comparing depression scores in people with T2DM. Whilst the overall sample included men, findings suggested that menopausal women scored significantly higher on depression scales than men (*p* < 0.02) and scored lower on perceived social support (*p* < 0.02). No significant differences were reported for pre‐menopausal women suggesting that menopausal women are at greater risk of depression with perceived social support potentially being a mediating factor. Furthermore, medication use was noted to have a negative correlation with depression symptoms in menopausal women but no correlation was found in pre‐menopausal women. However, it is pertinent to note that the broader literature highlights women living with diabetes experience higher rates of depression and anxiety regardless of reproductive stage^42^, ^43^ with women with T1DM more likely to have a prescription for anxiety/depression or diabetes distress than men.^43^ Therefore, it is suggested that the menopausal transition is an additional psychological stressor for an already vulnerable population.

One article exploring menopausal symptom severity and the association with glucose variability in women with T2DM found anxiety was more prevalent in those with elevated haemoglobin A1C (HbA_1c_) values.^31^ This reflects the findings from the included qualitative papers, which reported anxiety over glycaemic control and misinterpreting symptoms of hypoglycaemia for night sweats was a significant concern for women^6^, ^41^ with health implications such as a higher risk for cardiovascular disease and osteoporosis being cited.^19^ Other reported overlaps of diabetes and psychological menopause symptoms amplifying distress were brain fog and poor memory.^31^

The empirical papers support the consensus review by Lambrinoudaki et al.^40^ which broadly states that psychological menopausal symptoms may be influenced by diabetes. The interplay was discussed in relation to the potential exacerbation of psychological distress caused by glycaemic variability and HbA_1c_ and body mass associations being a further risk for distress.^31^, ^35^, ^40^ The authors also note the potential for early onset menopause in women with diabetes could increase psychological symptom burden due to prolonged hormonal deficiency.^40^

The typology of evidence (Table 4) for psychological symptoms in menopausal women with diabetes is dominated by Level 3–4 cross‐sectional studies, which consistently show elevated rates of depression, anxiety and reduced psychosocial well‐being in women with T2DM compared with women without diabetes. These findings are complemented by Level 3 qualitative studies demonstrating the unique emotional burden experienced by women with T1DM, particularly around symptom confusion and fear of hypoglycaemia. Interventional evidence (Level 2) indicates that behavioural counselling can reduce diabetes distress and improve emotional well‐being. Expert consensus supports these findings, suggesting that diabetes may amplify psychological menopausal symptoms through metabolic instability and prolonged hormonal deficiency. Overall, the evidence indicates that psychological symptoms are more severe and more disruptive in women with diabetes, although causality cannot be inferred due to the predominance of cross‐sectional designs.

**TABLE 4:** Typology of evidence—psychological symptoms.

Study	Categorisation	CEBM level^a^	Diabetes type	Population/context	Key psychological findings
Hasan et al.^29^	Empirical—Quantitative (cross‐sectional)	Level 4	T2DM	320 women with T2DM vs. controls	Higher depression and anxiety in T2DM; psychosocial factors strongest predictors of low QoL
Malacara et al.^25^	Empirical—Quantitative (cross‐sectional)	Level 4	T2DM	51 T2DM vs. 49 controls	Higher anxiety, depression and ‘empty nest syndrome’ in T2DM
Mezones‐Holguin et al.^10^	Empirical—Quantitative (cross‐sectional)	Level 4	T2DM	165 post‐menopausal women	Depression associated with sexual dysfunction; no diabetes‐specific difference
Rouen et al.^31^	Empirical—Quantitative (cross‐sectional)	Level 4	T2DM	327 female veterans	No group differences in depression/anxiety; higher anxiety in women with poor glycaemic control
Zimny et al.^35^	Empirical—Quantitative (cross‐sectional)	Level 3	T2DM	243 peri‐menopausal women	Lower psychosocial functioning in T2DM; ethnic differences in depression/anxiety
Florez et al.^37^	Empirical—Quantitative (cross‐sectional)	Level 3	T2DM	96 adults with T2DM	Menopausal women had higher depression, lower social support and lower adherence
Tirupathi et al.^38^	Empirical—Quantitative (cross‐sectional)	Level 3	T2DM	Post‐menopausal women	Higher psychological distress linked to diabetes duration
Parsa et al.^32^	Interventional (quasi‐experimental)	Level 2	T2DM	80 menopausal women	Counselling reduced diabetes distress and improved emotional well‐being
Mackay et al.^6^	Empirical—Qualitative	Level 3 qualitative	T1DM	10 women with T1DM	Fear, anxiety, emotional upheaval due to symptom confusion with hypoglycaemia
Forde et al.^41^	Empirical—Qualitative/PPI	Level 3 qualitative	T1DM/T2DM	184 women	High psychological distress; feeling overwhelmed by dual burden of diabetes + menopause
Lambrinoudaki et al.^40^	Expert consensus/Narrative review	Level 5	T1DM/T2DM	Global review	Diabetes may exacerbate psychological symptoms via glycaemic variability and metabolic stress
Slopien et al.^9^	Expert consensus	Level 5	T1DM/T2DM	European consensus	Diabetes increases vulnerability to psychological menopausal symptoms

Abbreviations: T1DM, type 1 diabetes mellitus; T2DM, type 2 diabetes mellitus.

^a^ Levels range from 1 (highest level of evidence) to 5 (lowest level of evidence).

#### 4.2.3 Physical symptoms

The most frequently reported physical symptoms of menopause in women with diabetes across studies were muscle and joint aches.^29^, ^30^, ^31^, ^34^, ^38^ Table 5 presents physical menopause symptom mapping.

**TABLE 5:** Physical menopause symptom mapping across studies.

Symptom	Source	Menopause status	Diabetes type
Musculoskeletal	Hasan et al.^29^	Post‐menopausal	T2DM
	Hasan et al.^30^	Post‐menopausal	T2DM
	Rouen et al.^31^	Post‐menopausal	T2DM
	Tirupathi et al.^38^	Post‐menopausal	T2DM
	Yang et al.^34^	Menopausal	T2DM
Weight gain	Mackay et al.^6^	Peri/menopausal	T1DM
	Malacara et al.^25^	Post‐menopausal	T2DM
	Parsa et al.^32^	Menopausal	T2DM
Fatigue	Mackay et al.^6^	Peri/menopausal	T1DM
	Zimny et al.^35^	Peri‐menopausal	T2DM
	Szadowska‐Szlachetka et al.^33^	Menopausal	T2DM
	Parsa et al.^32^	Menopausal	T2DM
	Yang et al.^34^	Menopausal	T2DM
Sleep disturbances	Mackay et al.^6^	Peri/menopausal	T1DM
	Rouen et al.^31^	Post‐menopausal	T2DM
	Zimny et al.^35^	Peri‐menopausal	T2DM
	Szadowska‐Szlachetka et al.^33^	Menopausal	T2DM
	Anderson et al.^36^	Peri/menopausal	T2DM
Accomplishing less (physical limitations)	Hasan et al.^30^	Post‐menopausal	T2DM
	Tirupathi et al.^38^	Post‐menopausal	T2DM
Digestive issues	Malacara et al.^25^	Post‐menopausal	T2DM

Abbreviations: T1DM, type 1 diabetes mellitus; T2DM, type 2 diabetes mellitus.

In studies that compared musculoskeletal symptoms with a control group, symptom prevalence was similar. However, severity was found to be higher in women with diabetes.^30^, ^31^ Suboptimal glycaemic control (HbA_1c_ >53 mmol/mol)^31^ and longer diabetes duration were linked to intensified musculoskeletal symptoms.^35^

Pertinent ethnic differences in musculoskeletal symptoms in menopausal women with and without TD2M were explored.^34^ Prevalence and severity scores were found to be significantly higher in non‐Hispanic white women with T2DM than those without, but no effect was found in Hispanic, Asian or African‐American groups. Therefore, menopause has been identified as a potential risk factor for increased musculoskeletal pain in white women with T2DM.

Six of the included studies did not report specific physical symptoms in women with diabetes.^10^, ^11^, ^24^, ^26^, ^37^, ^39^

The typology of evidence (Table 6) examining physical and musculoskeletal symptoms in menopausal women with diabetes is dominated by Level 3–4 empirical quantitative studies, which consistently demonstrate that while the *prevalence* of symptoms such as joint pain, muscle aches, fatigue and sleep disturbance is similar between women with and without diabetes, the severity and functional impact are significantly greater in women with T2DM. These findings are reinforced by methodological work validating QoL instruments in diabetes populations, which identifies the physical domain—including aching joints, low energy and reduced physical functioning—as a major contributor to symptom burden.^30^ Evidence also indicates that glycaemic control, BMI and diabetes duration are key modifiers of symptom severity, with poorer metabolic profiles associated with worse musculoskeletal outcomes. Qualitative evidence from women with T1DM adds depth to this picture, highlighting the compounding effects of fatigue and physical exhaustion within the context of intensive glucose monitoring. Expert consensus further supports the view that diabetes accelerates age‐related musculoskeletal decline, potentially amplifying the physical challenges of the menopausal transition. Collectively, the evidence suggests that physical symptoms are not inherently more common in diabetes but are more severe, more disruptive and more strongly shaped by metabolic factors, underscoring the need for integrated metabolic and menopausal care.

**TABLE 6:** Typology of evidence—physical and musculoskeletal symptoms.

Study	Categorisation	CEBM level^a^	Diabetes type	Population/context	Key findings
Yang et al.^34^	Empirical—Quantitative (cross‐sectional)	Level 3	T2DM	164 midlife women from 4 racial groups (62 with T2DM)	Women with T2DM had higher number and severity of musculoskeletal symptoms; ethnic differences observed (significant only in non‐Hispanic white women)
Rouen et al.^31^	Empirical—Quantitative (cross‐sectional)	Level 4	T2DM	327 post‐menopausal female veterans	Joint pain/stiffness highly prevalent; severity increased with poor glycaemic control (HbA1c >53 mmol/mol)
Hasan et al.^30^	Methodological (validation study)	Level 4	T2DM	215 women with T2DM (Malaysia)	MENQOL physical domain validated; physical symptoms (aches, low energy) strongly contributed to QoL burden
Hasan et al.^29^	Empirical—Quantitative (cross‐sectional)	Level 4	T2DM	320 women with T2DM vs. controls	Musculoskeletal symptoms more severe in T2DM; prevalence similar across groups
Zimny et al.^35^	Empirical—Quantitative (cross‐sectional)	Level 3	T2DM	243 peri‐menopausal women (116 with T2DM)	T2DM negatively impacted physical functioning, especially with higher BMI
Tirupathi et al.^38^	Empirical—Quantitative (CROSS‐SECTIONAL)	Level 3	T2DM	Post‐menopausal women with T2DM	Longer diabetes duration associated with greater musculoskeletal symptom severity
Parsa et al.^32^	Interventional (quasi‐experimental)	Level 2	T2DM	80 menopausal women with T2DM	Group counselling improved physical functioning and reduced symptom interference
Mackay et al.^6^	Empirical—Qualitative (interviews)	Level 3 qualitative	T1DM	10 women with T1DM	Fatigue and physical exhaustion were major concerns; exacerbated by increased glucose monitoring
Lambrinoudaki et al.^40^	Expert consensus/Narrative review	Level 5	T1DM/T2DM	Review of menopause in diabetes	Diabetes accelerates age‐related musculoskeletal decline, potentially worsening physical frailty during menopause

Abbreviations: T1DM, type 1 diabetes mellitus; T2DM, type 2 diabetes mellitus.

^a^ Levels range from 1 (highest level of evidence) to 5 (lowest level of evidence).

#### 4.2.4 Vasomotor symptoms

Papers reporting vasomotor symptoms are mapped in Table 7.

**TABLE 7:** Vasomotor symptom mapping.

Symptom	Source	Menopause status	Diabetes type
Hot flushes	Mackay et al.^6^	Peri/menopause	T1DM
	Hasan et al.^30^	Post‐menopause	T2DM
	Rouen et al.^31^	Post‐menopause	T2DM
	Zimny et al.^35^	Peri‐menopause	T2DM
	Parsa et al.^32^	Menopause	T2DM
	Yang et al.^34^	Menopause	T2DM
Night sweats	Mackay et al.^6^	Peri/menopause	T1DM
	Hasan et al.^30^	Post‐menopause	T2DM
	Rouen et al.^31^	Post‐menopause	T2DM
	Zimny et al.^35^	Peri‐menopause	T2DM
	Parsa et al.^32^	Menopause	T2DM
	Yang et al.^34^	Menopause	T2DM
Palpitations	Mackay et al.^6^	Peri/menopause	T1DM

Abbreviations: T1DM, type 1 diabetes mellitus; T2DM, type 2 diabetes mellitus.

Across the included studies, vasomotor symptoms—hot flushes, night sweats and palpitations—were reported in both qualitative and quantitative research, though the strength and direction of evidence varied by diabetes type and study design. Six studies found no consistent difference in vasomotor symptom prevalence or severity between women with and without T2DM,^6^, ^29^, ^30^, ^31^, ^32^, ^35^ although Hasan et al. noted differing patterns of hot flushes and night sweats. Rouen et al. observed greater anxiety and sleep disturbance in women with suboptimal glycaemic control, symptoms overlapping with vasomotor distress. In contrast, qualitative evidence from women with T1DM^6^ described ‘symptom mimicry’, where vasomotor sensations resembled hypoglycaemia, prompting anxiety and frequent glucose checks. Interventional evidence suggested behavioural counselling reduced interference from vasomotor symptoms without altering frequency.^32^ Overall, vasomotor symptoms do not appear biologically more severe in diabetes, but their clinical impact is heightened—particularly in T1DM by symptom overlap and psychological uncertainty. Table 8 presents the typology of evidence across studies exploring vasomotor symptoms.

**TABLE 8:** Vasomotor typology of evidence.

Study	Categorisation	CEBM level^a^	Diabetes type	Population/context	Key vasomotor findings
Rouen et al.^31^	Empirical—Quantitative (cross‐sectional)	Level 4	T2DM	327 post‐menopausal female veterans (USA)	No significant difference in prevalence or severity of hot flushes/night sweats between women with and without diabetes
Yang et al.^34^	Empirical—Quantitative (cross‐sectional)	Level 3	T2DM	164 midlife women (62 with T2DM)	Vasomotor symptoms did not differ significantly by diabetes status; musculoskeletal symptoms were worse in T2DM
Hasan et al.^30^	Methodological (validation study)	Level 4	T2DM	215 post‐menopausal women with T2DM (Malaysia)	MENQOL vasomotor domain valid; vasomotor symptoms associated with depression, but diabetes not a significant covariate
Hasan et al.^29^	Empirical—Quantitative (cross‐sectional)	Level 4	T2DM	320 women with T2DM vs. controls	Differences in *patterns* of vasomotor symptoms (hot flushes, night sweats), but no difference in overall MENQOL vasomotor scores
Zimny et al.^35^	Empirical—Quantitative (cross‐sectional)	Level 3	T2DM	243 peri‐menopausal women (Poland)	Vasomotor symptoms present but overshadowed by psychosocial and physical QoL issues in diabetic women
Parsa et al.^32^	Interventional (quasi‐experimental)	Level 2	T2DM	80 menopausal women with T2DM (Iran)	Group counselling reduced interference of vasomotor symptoms with daily life; metabolic markers improved
Mackay et al.^6^	Empirical—Qualitative (interviews)	Level 3 qualitative	T1DM	10 women with T1DM (Scotland)	Vasomotor symptoms often mimicked hypoglycaemia, causing anxiety and increased glucose checking
Slopien et al.^9^	Expert consensus	Level 5	T1DM/T2DM	European consensus on midlife health	Notes vasomotor symptoms as common in menopause; does not identify diabetes‐specific differences

Abbreviations: T1DM, type 1 diabetes mellitus; T2DM, type 2 diabetes mellitus.

^a^ Levels range from 1 (highest level of evidence) to 5 (lowest level of evidence).

#### 4.2.5 Age of menopausal onset

Earlier age of onset of menopause in those with T2DM is not consistently reported across findings.

Table 9 presents the typology of evidence across all papers which discussed the age of menopause in relation to diabetes.

**TABLE 9:** Typology of evidence: risk of early menopause in diabetes.

Study	Categorisation	CEBM level^a^	Diabetes type	Population/context	Key findings (menopause timing)
Yoshida et al.^39^	Empirical—Quantitative (longitudinal cohort)	Level 2	T2DM	9374 women (ARIC, MESA, JHS)	Higher prevalence of early menopause (<45 years) in T2DM; did not measure mean menopausal age
Lopez‐Lopez et al.^27^	Empirical—Quantitative (cross‐sectional)	Level 4	T2DM	404 women with T2DM vs. 409 controls	No significant difference in median menopausal age (49.8 vs. 49.6 years)
Malacara et al.^25^	Empirical—Quantitative (cross‐sectional)	Level 4	T2DM	51 T2DM vs. 49 controls	Earlier mean menopausal age in T2DM (*p* = 0.045); small sample, limited adjustment
Rouen et al.^31^	Empirical—Quantitative (cross‐sectional)	Level 4	T2DM	327 female veterans	No significant difference in menopausal age (*p* = 0.13); high BMI may mask early onset
Hasan et al.^30^	Empirical—Quantitative (cross‐sectional)	Level 4	T2DM	215 post‐menopausal women with T2DM	No significant difference in menopausal age
Hasan et al.^29^	Empirical—Quantitative (cross‐sectional)	Level 4	T2DM	320 women with T2DM vs. controls	No significant difference in menopausal age
Fang et al.^28^	Empirical—Quantitative (cross‐sectional)	Level 4	T2DM	1000+ Chinese women	No significant difference (*p* = 0.528)
Mezones‐Holguin et al.^10^	Empirical—Quantitative (cross‐sectional)	Level 4	T2DM	191 Peruvian women	No significant difference (*p* = 0.53)
Lambrinoudaki et al.^40^	Expert consensus/Narrative review	Level 5	T1DM/early‐onset T2DM	Review of ovarian ageing in diabetes	Suggests earlier menopause (1–2 years) in T1DM and early‐onset T2DM; based on mechanistic and small‐study evidence
Slopien et al.^9^	Expert consensus	Level 5	T1DM/T2DM	European consensus on midlife health	T1DM associated with earlier menopause; evidence for T2DM inconsistent and influenced by obesity

Abbreviations: T1DM, type 1 diabetes mellitus; T2DM, type 2 diabetes mellitus.

^a^ Levels range from 1 (highest level of evidence) to 5 (lowest level of evidence).

Across the included studies, evidence on whether women with diabetes experience earlier menopause is inconsistent and varies by diabetes type and study design. Most empirical studies (Level 3–4) reported no significant difference in menopausal age between women with and without T2DM.^27^, ^29^, ^30^, ^31^ One small and now outdated study found an earlier menopausal age in those with T2DM; however, its lack of adjustment for key confounders limits interpretation.^25^ In contrast, expert reviews (Level 5) suggest that T1DM and early‐onset T2DM may be associated with earlier ovarian ageing, potentially through autoimmune, microvascular or hyperglycaemic mechanisms. Limited epidemiological evidence, such as the pooled analysis by Yoshida et al.^39^ (Level 2), indicates a higher prevalence of early menopause in women with T2DM, though without detailed adjustment for adiposity or quantification of mean menopausal age. Overall, the most robust empirical evidence does not support earlier menopause in T2DM, whereas suggestive but low‐quality evidence points to a possible increased risk in T1DM and early‐onset T2DM.

### 4.3 Diabetes and menopause management and help seeking

Findings indicate that women accessed both formal and informal support, but distinctions were apparent. Formal support was predominantly accessed to address physical symptoms rather than mental health challenges, and was mainly provided by healthcare providers. Two studies described how interactions with health care providers, such as GPs and diabetes care teams, were often negative; HCPs lacked the necessary evidence base to adequately support them and often dismissed their concerns.^6^, ^41^

There was the suggestion that the management of diabetes and menopause was an unmet need within current healthcare services due to inadequate inclusion within guidelines, participant's education or routine clinical practice.^41^ The lack of information for women with diabetes regarding what to expect during menopause was discussed, including associated symptoms, where to seek help on adjusting insulin dosages, and advice on effective menopause management strategies. They reported the need for a more holistic approach to care. The result of the gap in guidance was an increased burden on women which impacted overall well‐being.^41^

Furthermore, barriers to seeking support included inadequate communication of needs due to fears of failure in relation to blood glucose management, specifically criticism surrounding loss of diabetes control due to HCP's minimising the hormonal effect of menopause on diabetes.^41^ Gender differences when speaking about their concerns were also discussed with the recognition that women would find it easier to discuss menopause with a female HCP.^6^

A key source of care for diabetes and menopause symptom management was the use of hormone replacement therapy (HRT). Evidence from the included studies suggests that women living with diabetes who are currently on HRT experience lower HbA_1c_ levels than women without HRT. Ferrara et al.^24^ compared HbA_1c_ levels in women with T2DM (drawn from the Northern California Kaiser Permanente Diabetes Registry) with and without HRT use. HRT was associated with 0.47 percentage points lower HbA_1c_ levels with no differences between women using opposed versus unopposed oestrogens. These findings were further analysed to assess if HbA_1c_ levels differed by the demographic variables of ethnicity and age with only age resulting in a significant difference (most significant difference in women aged 50–59 years, intermediate in women aged 60–69 years, and smallest in women aged ≥70 years). Ferrara et al. noted that women using HRT were found to be younger, leaner, better educated and likely to be non‐Hispanic white. In addition, it is salient to note that insulin treatment vs. oral hypoglycaemic agent was associated with a significant increase in HbA_1c_. The potential for HRT to improve glycaemic control was further evidenced in the review papers which concluded that HRT might improve glycaemic control in women with pre‐existing diabetes.^9^, ^40^

The use of HRT to improve glycaemic control was reported as a significant concern for women living with diabetes during menopause. Glycaemic management was reported as a major goal in women with diabetes during the menopausal journey,^6^ as poor glycaemic control is associated with long‐ term effects on cardiovascular health.

Despite this, Davies and Barnett^26^ highlighted a lack of knowledge and awareness of HRT in relation to perceived risk and benefits in women with diabetes. They reported just 43% of pre‐menopausal, 37% peri‐menopausal and 20% post‐menopausal women thought HRT was safe for those with diabetes. Notably, 15% of the peri‐menopausal women and 13% pre/post‐menopausal women thought HRT might improve their diabetes control. Whilst the Davies and Barnett paper may not reflect contemporary perceptions surrounding the risks, the more recent paper by Mackay et al.^6^ suggested that there was evidence of preconceived ideas around the contraindications of HRT within their sample.

Limited interventional research explored diabetes management and menopausal symptom management, with only two identified for inclusion.^36^ One feasibility study completed a 12‐week eHealth intervention for women's health in a sample of midlife women living with T2DM and observed significant improvements in menopausal symptoms and sleep symptoms from baseline to programme completion. The authors adopted the social cognitive theory^44^ as their theoretical underpinning. They targeted self‐efficacy and additional aspects relating to the Theory of Planned Behaviour,^45^ namely perceived behavioural control, to promote health behaviour change through a wellness programme. While the authors do not list specific menopause symptoms, it indicates that the programme had a positive impact across all domains as scored by the Greene Climacteric Scale.^36^, ^46^ Importantly, the findings also found the intervention resulted in a significant reduction in diabetes distress and improved health related QoL; therefore, further indicating the intervention's effectiveness for women living with diabetes during menopause.

Similarly, findings from a paper exploring the effectiveness of group counselling on self care behaviours (exercise, diet, foot behaviours) and metabolic indicators (e.g. fasting blood glucose and HbA_1c_) in menopausal women with diabetes reported group counselling promoted self care behaviours and metabolic indices.^32^

Exploration of informal support was limited in the included papers. However, Mackay et al.^6^ suggest that social support systems with peer support provide opportunities for women living with diabetes to gain knowledge and coping strategies from other women who have experience of diabetes and menopause.^6^

Although cultural comparisons were not undertaken in any study, synthesis across settings indicates a common pattern: women with diabetes receive little formal menopause support regardless of context. UK qualitative studies reveal particularly stark gaps in HCP communication and HRT guidance, while international papers similarly lack evidence of structured menopause care. This points to a systemic, cross‐cultural deficit in integrated menopause–diabetes support.

### 4.4 Severity of menopause symptoms and impact on quality of life

Quality of life (QoL) and menopausal symptom severity were assessed using validated instruments in five of the included papers.^29^, ^30^, ^31^, ^32^, ^38^

Whilst not part of the current review's initial aims and objectives, what was notable during data extraction and evidence synthesis was the heterogeneity of the scales used to assess QoL in relation to menopausal symptoms and no quantitative QoL studies were identified for women with T1DM. This made conclusions regarding the impact of menopause in women with diabetes difficult to assess.

One study (Level 4) explored the validity of the Menopause Specific Quality of Life Scale (MENQOL) in women with diabetes.^30^ Reported a significant association between psychosocial domain scores and depression, however, diabetes was not a significant covariate when compared with domain scores on the Short Form Health Survey (SF‐12).^30^ Two studies utilised the MENQOL as a measurement for menopause QoL in women with T2DM as their primary aim.^29^, ^38^ Both agree that T2DM is a risk factor for lower menopausal QoL, with higher symptom burdens in vasomotor, physical, psychosocial, and sexual domains. Women with T2DM have an increased risk for depression, anxiety, and physical complaints (e.g. aches, hot flushes) than controls, leading to lower overall QoL. Key contributing factors include hormonal changes interacting with T2DM and exacerbated symptoms. Body composition also correlated to worse QoL, especially in peri‐menopausal stages. Psychological issues like depression are more prevalent and amplify QoL decline. The demographic and cultural contexts (Malaysia, Poland, India) demonstrate similar patterns suggesting global relevance.

It was noted by Rouen et al.^31^ that women with suboptimal glucose control demonstrated more severe anxiety and sleep disturbances than those with better glucose control, more severe mood swings than women without diabetes, and more intense headaches compared with both groups. Total Menopausal Symptoms Severity (MSL) scores were higher in women with diabetes with sub‐optimal glucose control compared with the other women with optimal control and those without diabetes. While no statistical association was explored, this descriptive observation highlights the possible link between glycaemic control and menopause symptom severity.

The intensity of menopause symptoms measured with the Batt‐Kupperman Menopausal Index scale was explored in relation to QoL scores on the WHOQOL‐BREF^33^, ^47^, ^48^ and the resulting impact of QoL was reported to be positively correlated with the evaluation of lower QoL by the respondents. However, the findings were analysed on the whole sample (*n* = 161) with only ten women living with diabetes. Therefore, findings from this study on whether diabetes has an increased impact on QoL cannot be considered for evidence of lower QoL in this review.

In contrast, no quantitative QoL evidence exists for women with T1DM. Instead, qualitative studies describe menopause as a period of marked glycaemic instability, emotional burden and reduced confidence in self management, although both studies were conducted exclusively with White UK women.^6^, ^41^ These gaps highlight the need for culturally sensitive research to understand how ethnicity, symptom experience and diabetes interact to shape menopausal QoL. Overall, moderate‐quality observational evidence and one quasi‐experimental study support an association between T2DM and reduced menopausal QoL, particularly in the context of poorer glycaemic control, while QoL impacts in T1DM remain largely unquantified and reliant on qualitative accounts.

### 4.5 Interplay of menopause and diabetes

Several papers measured both menopausal symptoms (via MENQOL, Greene, Blatt‐Kupperman or symptom checklists) and HbA_1c_ or fasting glucose. Some performed correlation or regression analyses between glycaemic control and symptom scores. While improved control was associated with fewer symptoms in some, all findings were non‐significant. None presented any statistical association between insulin resistance (measured or estimated via Homeostatic Model Assessment for Insulin Resistance (HOMA‐IR), fasting insulin, Quantitative Insulin Sensitivity Check Index (QUICKI), or any other index) and menopausal symptom severity, frequency or scores (MENQOL, Greene, Blatt‐Kupperman or symptom checklists). In contrast, qualitative and PPI evidence highlighted perceived, but not objectively measured, links between menopause and glucose variability, including difficulty distinguishing vasomotor symptoms from hypoglycaemia and increased emotional burden around glycaemic management. Overall, the evidence base indicates that while women frequently report interactions between menopause and diabetes control, empirical studies have not yet established a physiological association between menopausal symptoms, glycaemic control or insulin resistance. Table 10 presents studies which reported factors associated with diabetes management.

**TABLE 10:** Diabetes management and menopause symptom severity.

Study	Measured glycaemic control (HbA1c/glucose)	Reported link between glycaemic control & symptom severity	Measured insulin resistance (HOMA‐IR/fasting insulin)	Reported link between Insulin resistance & symptom severity	Diabetes type
Rouen et al.^31^	Yes (HbA1c)	*No statistical association tested*. Descriptive differences only; women with poorer control appeared to have higher symptom scores	No	Not assessed	T2DM
Parsa et al.^32^	Yes (HbA1c)	No correlation reported; HbA1c did not differ significantly between high‐ and low‐symptom groups	No	Not assessed	T2DM
Tirupathi et al.^38^	Yes (HbA1c)	HbA1c reported descriptively; *no analysis* linking HbA1c to Greene scores	No	Not assessed	T2DM
Hasan et al.^30^	Yes (HbA1c)	HbA1c reported; *no statistical association* with MENQOL domain scores	No	Not assessed	T2DM
Hasan et al.^29^	Yes (HbA1c)	HbA1c reported; *no statistical association* with MENQOL or psychological domains	No	Not assessed	T2DM
Malacara et al.^25^	Yes (fasting glucose & insulin)	No analysis linking glucose or insulin to menopausal symptoms	Yes (fasting insulin)	Not assessed	Mixed (mostly T2DM)

Abbreviation: T2DM, type 2 diabetes mellitus.

## 5 DISCUSSION

This review has addressed the research question: ‘What is known from the literature about women's experiences of living with diabetes and menopause and its impact on their quality of life?’ The results have illustrated that these women experience a range of menopausal symptoms, often more severely than those without diabetes. Coupled with a lack of clarity about care pathways or means of support, these experiences have a negative impact on QoL.

### 5.1 Diabetes and QoL

Significant gaps in research and clinical guidelines for the management of these two conditions remain with negative effects on adequate support and diabetes outcomes as well as women's overall QoL. This review highlighted the significant lack of interventional research to improve QoL in this population and reflects the urgent need for further research in this area. Although sparse, evidence to support the impact of potential interventions with improvements on menopause symptoms and diabetes self care behaviours was demonstrated^6^ with diabetes distress linked to self‐efficacy.

This indicates that providing women with strategies to increase a sense of control over their diabetes can reduce diabetes distress and menopause‐related symptoms. By exploring effective interventions from which strategies can be developed and adopted by current diabetes services, women can be better supported through this phase of their life.

The need for effective tools for assessing symptoms and QoL in this population is apparent. Validated instruments for use during the menopause use a variety of items relating to symptom prevalence or severity and QoL factors. Indeed, the Blatt‐Kupperman scale has been criticised for its lack of exploration into symptoms such as vaginal dryness and loss of libido and issues with poorly defined terminology highlighted.^49^

Additionally, current literature suggests that there are robust instruments available to assess QoL in menopause with a good coverage of symptoms (if amalgamated); however, none address all aspects of HRT as an influencing variable on QoL.^50^ It has been suggested that current measures are modified or a new instrument developed.^46^ The most recent and widely used instrument within the included articles was developed in 1998.^46^ With contemporary research on menopause becoming more prevalent, eliciting more awareness of the complexities of the individual experience of menopause, particularly when living alongside diabetes, the need for an up‐to‐date measure is evident.

Similarly, while there was evidence to suggest that women living with diabetes are at a greater risk of sexual dysfunction, consideration in relation to cultural validation of FSD cut‐off scores is required. The Female Sexual Function Index (FSFI) was originally validated in an American population with a cut‐off score of 26.55.^51^ Since its development it has been validated in other populations including Egypt^52^ Spain^53^ and those reported within this review^10^, ^11^, ^28^ with variations in cut‐off scores ranging from 25 to 28.1. This makes comparison of outcomes and global consensus of diabetes related FSD in menopause difficult, creating a fragmented international research landscape, impacting prevalence, risk factor analysis and clinical applicability. Studies addressing this methodological disparity are required to provide global insights into diabetes, menopause and FSD interactions.

Qualitative evidence highlights a parallel challenge, in relation to QoL. Women report being poorly informed about menopause, with limited guidance on managing both menopause and diabetes simultaneously. Many describe their concerns as dismissed or minimised by healthcare professionals, reflecting broader gendered communication gaps in chronic disease management.^54^ This lack of integrated, person‐centred support may delay symptom recognition, reduce help‐seeking and undermine confidence in navigating the menopause transition.

### 5.2 Menopause symptoms

Evidence for earlier menopause in women with diabetes is weak and inconsistent. Across multiple large cross‐sectional studies, T2DM shows no meaningful difference in menopausal onset, despite varied populations and measurement approaches. The single study suggesting earlier menopause in T2DM is small, outdated and unadjusted, offering little credible support. Limited longitudinal evidence indicates a higher prevalence of early menopause in T2DM, but without robust adjustment for adiposity or reporting of mean age. Claims of earlier menopause in T1DM or early‐onset T2DM stem largely from low‐quality research or expert opinion, not strong epidemiological data. Overall, the most reliable evidence refutes earlier menopause in T2DM, while the signal in T1DM remains speculative and urgently requires rigorous longitudinal study.

Across the reviewed studies, women with diabetes consistently reported more severe menopausal symptoms than women without diabetes, particularly in the psychological, vasomotor, musculoskeletal and genitourinary domains. Quantitative studies demonstrated higher overall symptom burden and poorer menopause‐related QoL in T2DM, including greater vasomotor and urogenital discomfort,^30^, ^35^ more intense sexual symptoms such as dyspareunia, reduced lubrication and lower FSFI scores^10^, ^11^, ^28^ and broader physical and emotional disruption.^31^ Studies focusing on FSD reported notably higher prevalence in women living with T2DM. Evidence from broader clinical reviews indicates that menopause and diabetes exert additive and mutually reinforcing effects on sexual function, as oestrogen deficiency reduces vaginal elasticity and lubrication while diabetes‐related microvascular and neuropathic changes further impair genital blood flow, sensation and arousal. This interaction creates additional burden in midlife, with women experiencing sexual difficulties more intensely, and with greater persistence than women without diabetes, a pattern consistently highlighted in clinical overviews of sexual health in those with diabetes.^55^ In addition to these biological interactions, the ongoing demands of diabetes self management, such as glucose monitoring, dietary restrictions, medication adherence and fear of complications, can further diminish sexual well‐being by increasing psychological distress, reducing spontaneity and reinforcing negative body image. These behavioural and emotional pressures operate alongside the physiological effects of oestrogen deficiency and diabetes‐related vascular and neuropathic damage, creating a cumulative burden that heightens the severity and persistence of FSD in menopausal women with T2DM. Furthermore, cultural expectations around modesty, gendered power dynamics and the stigma associated with discussing sexual well‐being create substantial barriers for many women, particularly those from ethnic minority backgrounds or living in low‐ and middle‐income contexts, where sexual health is often considered a private or taboo topic. These cultural pressures intersect with healthcare‐system barriers, such as limited consultation time, lack of clinician training and assumptions that women will not wish to discuss sexual concerns. Ths can result in missed opportunities for assessment and support for menopausal women with diabetes.^56^ Qualitative evidence further highlighted the compounded severity of symptoms in T1DM, with women describing amplified fatigue and mood disturbance that interfered with diabetes self management.^6^ Despite heterogeneity in measurement tools, the direction of findings was consistent: diabetes appears to intensify menopausal symptom severity, contributing to a more challenging and disruptive menopausal experience.

### 5.3 Blood glucose regulation

In T2DM, moderate‐quality quantitative evidence links post‐menopausal status to higher HbA1c and reduced insulin sensitivity^24^, ^25^ while behavioural interventions can improve glycaemic outcomes.^32^ In contrast, evidence for T1DM is almost entirely qualitative, with women reporting marked glycaemic instability, fluctuating insulin requirements and difficulty distinguishing vasomotor symptoms from hypoglycaemia^6^, ^41^ These qualitative studies report feelings of frustration, fear and “loss of control,” compounded by the perception that healthcare professionals lack menopause‐specific guidance for women with diabetes. Together, these factors can substantially impair emotional well‐being, daily functioning and overall QoL during the menopausal transition.

### 5.4 HRT in diabetes

Research focusing on HRT use in post‐menopausal women with T2DM was associated with a statistically significant increase in insulin sensitivity, demonstrating the link between declining hormones in menopause and insulin resistance.^57^, ^58^ It is salient to note that these studies focus on post‐menopausal women living with T2DM. There is no empirical evidence evaluating the impact of HRT on glycaemic control in women with diabetes, highlighting a major gap in the literature and an urgent research priority.

Since undertaking this review, additional research into HRT for women with diabetes has been published that supports the suggestion that HRT may contribute to better glycaemic control, improved lipid metabolism and enhanced insulin sensitivity for women with T2DM.^59^ However, despite these potential benefits, clinical guidelines advocate for ‘cautious use’ in strictly screened patients, emphasising early intervention (within 10 years of menopause) and personalised risk assessments for cardiovascular disease and venous thromboembolism (ref). The 2026 NICE guidelines for menopausal symptom management in women with DM have not changed since first published in 2015. These guidelines state HRT in women with T2DM should be considered after taking comorbidities into account and seeking specialist advice if needed. Conversely, the evidence specifically for women with T1DM remains limited. A 2010 systematic review noted available evidence for healthcare professionals was ‘vague’.^60^ As of May 2026, the significant evidence gap endorses the need for large scale RCTs in the use of HRT specifically in a T1DM population.

### 5.5 Limitations/methodological considerations

Scoping reviews provide a systematic and transparent method of identifying a broader exploration of the evidence base than is achievable with other forms of review.^61^ The current review has provided valuable insights into an under explored subject; however, limitations of the individual studies and their methodology should be acknowledged.

There was a scarcity of evidence around women's personal experience of *living with* diabetes during the menopause highlighting a significant gap within the literature that should be addressed. However, it is important to note that while the included qualitative and PPI papers^6^, ^41^ provide salient observations, constraints regarding the weight of their evidence need consideration. Recruitment methods that utilised online social media are more susceptible to bias with individuals who are more active in online diabetes spaces; therefore, they may not represent the views of all women living with diabetes. Similarly, the paper by Mackay et al.^6^ included a sample of 10 white women from one hospital clinic in the United Kingdom. While the small sample size was suitable for a grounded theory approach, the findings are specific to this single site. Furthermore, the qualitative findings explored the experiences of women with T1DM and therefore may not reflect the experience of women living with T2DM.

Cross‐sectional research made up a significant proportion of studies included in this review and is therefore more susceptible to bias and lacks the ability to determine a cause–effect relationship.^62^ Equally salient was the heterogeneity in the operationalisation of the terms peri‐menopause, menopause and post‐menopause across the included studies which made comparison and synthesis of the data more challenging. The age of women in the studies varied widely; for example, women over 40 years designated post‐menopause^10^ while others suggesting 45–72 years as menopausal.^25^ The definition of the peri‐menopausal period was seen in one study to include women aged between 45 and 65 years yet reporting that 74% of their sample had not been menstruating.^35^ Moreover, they reported the average time since last menstruation was 9.79 years, which, according to guidelines should be defined as post‐menopausal.^1^ These inconsistencies were seen throughout the included papers with self‐reported status a further limitation. For clarity, future research should consider how menopausal status is defined to ensure accurate exploration into the nuances and challenges women might face throughout each menopausal phase.

The heterogeneity in measurement of QoL, menopausal status and menopausal symptoms throughout the included papers resulted in significant challenges for meaningful evidence synthesis and robust conclusions around the impact of living with diabetes during the menopause. These methodological inconsistencies reduce the reliability and precision of cross‐study comparisons, and the conclusions drawn should therefore be interpreted as indicative rather than definitive. Nonetheless, the consistency in the *direction* of findings across diverse measures strengthens confidence in the overarching patterns identified, while underscoring the need for standardised outcome measures in future research.

Furthermore, women with T1DM are strikingly under‐represented in the menopause literature, with only a small number of qualitative or consensus‐based papers available and no epidemiological studies or interventional trials. The limited evidence that does exist highlights distinct challenges, including glycaemic instability and confusion between vasomotor symptoms and hypoglycaemia^6^ yet these insights remain anecdotal and derived from very small samples. Importantly, no menopause‐specific clinical guidelines exist for women with T1DM, and major bodies such as NICE, American Diabetes Association and the Endocrine Society provide no tailored recommendations for this group. As a result, the evidence base is insufficient to characterise symptom patterns, metabolic changes or support needs in T1DM, representing one of the most significant gaps identified in this review.

### 5.6 A bidirectional hypothesis

While the review has evidenced the negative impact of the menopause on women with diabetes, the relationship between diabetes and menopause is hypothesised as bidirectional, with each condition shaping the trajectory, symptom burden and metabolic profile of the other and impacting on overall QoL.

Literature which did not meet the criteria for this review provides support for the hypothesised bidirectional relationship between diabetes and menopause hypothesis includes Speksnijder et al.^58^ who report that menopause‐related oestrogen deficiency may adversely affect glucose regulation, while oestrogen‐containing menopausal hormone therapy produces neutral‐to‐beneficial effects on glycaemic control, significantly lowering HbA1c and fasting glucose in post‐menopausal women with diabetes, with evidence strongest for T2DM and insufficient for T1DM. Overall, the hypothesised bidirectionality is supported in T2DM but remains largely experiential and unquantified in T1DM, underscoring the need for dedicated mechanistic and longitudinal research.

In conclusion, this scoping review shows that women with diabetes experience a wide range of menopausal symptoms, often with greater severity and impact on QoL than women without diabetes, particularly in the psychological, musculoskeletal and genitourinary domains. Evidence was dominated by studies of women with T2DM, with minimal research addressing the experiences of those with T1DM. Despite frequent reporting of HbA1c and glucose measures, few studies examined their relationship with menopausal symptoms, and none assessed insulin resistance or the effects of hormone therapy on glycaemic outcomes, leaving key metabolic questions unanswered. Methodological limitations, including the predominance of cross‐sectional designs, heterogeneous symptom measures, small samples and inconsistent reporting of menopausal status, restricted comparability and causal interpretation. These gaps highlight the need for robust longitudinal and mechanistic research, alongside integrated clinical approaches that better support women managing both diabetes and menopause.

## AUTHOR CONTRIBUTIONS

This review was conceptualised and designed by VC, FH, LK and RB. The first author (RB) conducted the searches. Article selection and data extraction were undertaken by five reviewers RB, VC, FH, LK, DM. RB prepared the manuscript. All authors were involved in the interpretation of data. The remaining authors reviewed and assisted with finalising the article. All authors provided final approval of the submitted version of the manuscript, and all agree to be accountable for all aspects of the work.

## FUNDING INFORMATION

This research is supported by Diabetes UK grant (23/000554).

## CONFLICT OF INTEREST STATEMENT

The authors declare no conflicts of interest.

## Supporting information


**File S1.** Preferred reporting items for systematic reviews and meta‐analyses extension for scoping reviews (PRISMA‐ScR) checklist.

**File S2.** Full electronic Search Strategy.

**File S3.** Characteristics and main findings of included studies examining menopause in women with T1DM and T2DM.

## Data Availability

Data supporting the findings of this study are available from the corresponding author upon reasonable request.
