# Supplementary material for: Impact of diabetes mellitus on the menopausal experience: A scoping review
Source: Diabet Med. 2026 Aug 22;43(10):e70420. doi: 10.1111/dme.70420 (PMC13643661; doi:10.1111/dme.70420)
Supplement: Supplementary file 1 [file DME-43-e70420-s001.docx]

Supplementary File 2. Full electronic Search Strategy

Top of Form

| Set | Search Statement |
| --- | --- |
| 1. | exp *Menopause/ |
| 2. | Perimenopause/ |
| 3. | Climacteric*. |
| 4. | Menopaus*.mp. |
| 5. | Peri?menopaus*. |
| 6. | exp *Diabetes Mellitus/ |
| 7. | *Diabetes Mellitus, Type 1/ |
| 8. | *Diabetes Mellitus, Type 2/ |
| 9. | Diabet*. |
| 10. | iddm. |
| 11. | niddm. |
| 12. | Insulin depend*. |
| 13. | Insulin?depend*. |
| 14. | Non insulin depend*. |
| 15. | T1d. |
| 16. | T2d. |
| 17. | Non?insulin depend*. |
| 18. | T1dm. |
| 19. | T2dm.mp. |
| 20. | Climacteric/ |
| 21. | 1 or 2 or 3 or 4 or 5 or 20 |
| 22. | 6 or 7 or 8 or 9 or 10 or 11 or 12 or 13 or 14 or 15 or 16 or 17 or 18 or 19 |
| 23. | 21 and 22 |

Bottom of Form
