# Supplementary material for: Impact of diabetes mellitus on the menopausal experience: A scoping review
Source: Diabet Med. 2026 Aug 22;43(10):e70420. doi: 10.1111/dme.70420 (PMC13643661; doi:10.1111/dme.70420)
Supplement: Supplementary file 3 [file DME-43-e70420-s003.docx]

Supplementary File 3. Characteristics and main findings of included studies examining menopause in women with T1DM and T2DM.

| Citation details | Country/ funding | Aim | Context | Participant details | Study Design | Analysis | T1DM/T2DM | Menopausal Status | Findings relevant to scoping review | Scales |
| --- | --- | --- | --- | --- | --- | --- | --- | --- | --- | --- |
| Malacara JM, Huerta R, Rivera B, Esparza S, Fajardo ME. Menopause in normal and uncomplicated NIDDM women: physical and emotional symptoms and hormone profile. *Maturitas*. 1997;28(1):35-45. doi:10.1016/s0378-5122(97)00051-0  In text citation number - 26 | Mexico  Funding:  CONACYT (Consejo Nacional de Ciencia y Tecnología) Mexico's federal agency for science, technology, and innovation. | To compare the physical characteristics, emotional symptoms and metabolic conditions of menopausal women with and without non-insulin dependent diabetes mellitus (NIDDM) | Mexican public – recruited via household visits and public areas | N = 100  (n= 51 T2DM; n = 49 no DM) aged 45-72 years  Diabetes diagnosed according to the NDDG criteria as they had at least two previous blood glucose tests higher than 11.1 mmol/l.  Diabetic patients had a mean of 7.7 + S.D. 8.6 years since diagnosis | Cross-sectional | characteristics of postmenopausal diabetic and nondiabetic women were compared with the Mann-Whitney U.  In the group of women with NIDDM the symptoms were compared for the subgroups with good or fair control ( < 11.1 mmol/l), vs the group with bad metabolic control of diabetes ( 2 11.1 mmol/l). In the group without NIDDM they compared the frequency of symptoms in subgroups with fasting blood glucose levels of < 5.5 mmol/l and with 5.5-7.8 mmol/l. The analysis of factors associated with physical and emotional symptoms was carried out with a multiple stepwise regression procedure with forward inclusion of variables  (pairwise deletion of missing data) taking as dependent variables depression, anxiety, NSSD and ENS.  Factors associated with DHEAS, cortisol, insulin/glucose indices and FSH were studied by means of stepwise multiple regression | T2DM | Natural Post Menopause: defined as amenorrhea for more than 12 months in women with previously regular cycles. | Mean age at menopause was earlier in the NT2DMgroup (45.7 vs. 48.0, P= 0.045).  Association of age at diagnosis of diabetes and age at menopause. A positive correlation was found (r = 0.308, P < 0.029).  Diabetic women had higher depression scores (P = 0.046) and ENS (P = 0.019).  Comparing symptoms in NIDDM patients with good and fair control (n = 18) vs those with bad metabolic control (blood glucose 2 1 I. 1 mmol/l) (n = 33), no significant differences were found.  symptoms in the subgroups of menopausal women without NIDDM, according to the fasting blood glucose, comparing the group with < 5.5 mmol/l (n = 27) and with 5.5-7.8 mmol/l (n = 22), no significant differences in the frequency of symptoms. | Modified Hamilton and Beth-Rafaelsen scale (HSD/MES)  The empty nest syndrome (ENS) was assessed with questions, to be answered with yes or no, taken from the DSM-III-R. |
| Davies PH, Barnett AH. Hormone replacement therapy in women with diabetes mellitus: A survey of knowledge of risks and benefits. *Practical Diabetes International*. 1998;15(3):78-81. doi:10.1002/pdi.1960150307  In text citation number - 27 | UK  Funding not reported | To assess knowledge and beliefs about HRT in diabetic women - awareness and use, sources of information and knowledge of benefits and risks | Women attending a UK diabetic clinic | Women of all ages (22-90 yrs) median age: n = 58  Seven women did not state their age but  otherwise completed the questionnaire satisfactorily  and the data they provided was analysed only for the group as a whole. | Cross-Sectional Survey | The group was analysed as a whole and then  divided into three sub-groups for further  comparison on the basis of age: premenopausal  women (aged **<**45 years), perimenopausal  women (aged 45-65 years) and  postmenopausal women (aged >65 years). | T1DM | n = 130 menopausal or  approaching the menopause (71 %) and 25 of  these were taking HRT (19%): 42 women  were aged <45 years (premenopausal group);  75 women were aged 45-65 years (perimenopausal  group); and 58 women were  aged *>65* years (postmenopausal group). | **Knowledge of benefits and risks of HRT**  49% of all respondents felt able to  answer one or more of the questions in this  section. 61% premenopausal; 55% perimenopause; 26% postmenopausal  **Perceived risks of HRT**  43% of premenopausal women thought HRT use  was safe for diabetics, falling to 37% in the  perimenopausal group and to 20% in the postmenopausal group.  No premenopausal woman thought that HRT could increase the risk of heart disease; 12% of the perimenopausal  group and 27% of the postmenopausal group thought this to be true.  No premenopausal women thought that HRT could worsen blood pressure;17% of the perimenopausal group and  20% of the postmenopausal group thought that it might.  HRT increases risk of cancer:  23% premenopausal  37% perimenopausal group and  40% postmenopausal  group.  **Perceived Benefits of HRT**  HRT might improve diabetes control in some way:  13% Pre/Post menopause  15% perimenopausal  HRT use can decrease the risk of osteoporosis:  80% of premenopausal women  83% of perimenopausal  women  73% postmenopausal women  **Key points**  *Postmenopausal HRT confers greater benefit to women at increased risk of*  *ischaemic heart disease, such as those with diabetes.*  *Awareness of the potential benefits and risks of HRT is poor in women with*  *diabetes.*    *Over two thirds of diabetic women described their knowledge of HRT as*  *inadequate.*  *63% thought HRT was not safe for diabetics.*  *Greater discussion concerning HRT would enable women with diabetes to make informed choices in the future.* | N/A |
| Lopez-Lopez R, Huerta R, Malacara JM. Age at menopause in women with type 2 diabetes mellitus. *Menopause (New York, NY)*. 1999;6(2):174-178.  In text citation number - 28 | Mexico  Funding:  CONACYT (Consejo Nacional de Ciencia y Tecnología)-  Mexico's federal agency for science, technology, and innovation. | To compare the age at menopause in women with T2DM and women without | Mexican Public and Mexican Hospitals | N = 172 menopausal women without DM  N = 207 women with T2DM | Cross-sectional | Status quo method with regression analysis with maximum likelihood | T2DM | Natural Menopause | Median age at menopause for whole group – 49.7 ± SD 1.7 years; without diabetes - 49.6 ± SD 1.6; 49.8 ± SD 1.7 yrs  Hot flushes: No significant difference | N/A |
| Ferrara A, Karter AJ, Ackerson LM, Liu JY, Selby JV. Hormone replacement therapy is associated with better glycemic control in women with type 2 diabetes: The Northern California Kaiser Permanente Diabetes Registry. *Diabetes care*. 2001;24(7):1144-1150.  In text citation number - 25 | USA  Funding: American Heart Association Western States Affiliate & SmithKline Beecham  Pharmaceuticals. | To examine whether HbA1c levels varied by current HRT among women with type 2 diabetes | Kaiser Permanente Medical Care Program (KPMCP) of Northern California (a group practice, prepaid health plan) | Women with type 2 diabetes n = 15,435  Aged 50-98 (mean 64.7, SD 6 8.7)  3,852 (25%) were currently using HRT before the HbA1c test. Among women currently using HRT, 62% were using unopposed oestrogens, 36% were using opposed oestrogen, and 2% were using progestins alone.  55% non-Hispanic whites, 14% non-Hispanic blacks, 12% Hispanics, 11% Asians, 4% “other” ethnic groups, and 4% with missing ethnicity data. | Observational Study | *t-*tests; Age-adjusted mean  levels of HbA1c and BMI by HRT were  computed and compared using analysis  of covariance. A generalized estimating  equation (GEE) model was constructed to  assess the association between HRT and  HbA1c level | T2DM | Not specified | In the assessment for whether association between HRT and HbA1c differed by ethnicity and age only HRT and age was statistically significant (*P* , 0.0001).  Women currently using HRT were younger, leaner, better educated, and more likely to be non-Hispanic whites  than women not using HRT  Mean HbA1c levels were significantly lower in women currently using HRT  than in women not using HRT, and these differences increased after adjusting for  age.  No differences in HbA1c level were observed between women using opposed oestrogens and women using unopposed oestrogens (*P* 5 0.48). Both women using oestrogens alone or oestrogens in combination with progestins had significantly lower HbA1c mean levels than women not using HRT (age-adjusted mean [SE] 8.0 [0.03] and 8.0 [0.04], respectively, vs. 8.5 [0.02]; *P* 5 0.0001).  analyses were repeated after excluding the 2,232 women  without continuous medication benefits, and similar differences in HbA1c levels were observed between current HRT users and nonusers (*P* 5 0.0001).  Association between current HRT and decreased  HbA1c levels remained statistically significant in all of the age strata, it was most significant in women aged 50–59 years, intermediate in women aged 60 – 69 years, and smallest in women aged ≥70  Associations remained significant after stratifying: type of hypoglycaemic therapy; ethnicity; education level; BMI; diabetes duration. Similar associations found when stratified by exercise.  HRT use was associated with 0.47-point lower HbA1c levels  Insulin treatment vs oral hypoglycaemic agent associated with significant increase in HbA1c – other predictors of increase include U.S. minority ethnic groups, obesity, and  longer diabetes duration. Significant direct association between SMBG practice less than once a week and HbA1c. | N/A |
| Mezones-Holguin E, Blumel JE, Huezo M, et al. Impact of diabetes mellitus on the sexuality of Peruvian postmenopausal. *Gynecological endocrinology : the official journal of the International Society of Gynecological Endocrinology*. 2008;24(8):470-474. doi:10.1080/09513590802273749  In text citation number - 10 | Peru  Funding not reported | To evaluate the sexual function in women complaining of both  conditions, postmenopausal and DM | Women attending hospital in Peru | Total: n= 72  n= 36 T2DM  n= 36 controls | Prospective study | t-tests, fishers, logistic regression | T2DM | Post-menopausal | T2DM women had a worse marital relationship  on a scale of 0 to 20 (15.1+1.9 vs. 13.4+2.9, p50.009), lower frequency of sexual intercourse per month (2.7+2.8 vs. 4.4+2.9, p50.01) and higher depression scores than the control group (11.5+5.6 vs. 8.9+4.7, p<0.03).  Diabetic women had worse scores than controls in all domains of the FSFI.  The prevalence of FSD in the DM group (FSFI  Score ≤26.55) was 75.0% vs. 30.6% in controls  (p<0.001). Among diabetics those with poor metabolic  control (Hb1Ac>7.0%) had worse total scores  in the FSFI than those with adequate metabolic  control (16.6+8.4 vs. 24.2+5.1, p<0.006).  After confounding factors – DM was still most important factor FSD (OR= 6.2,  95% CI 2.0–19.6, p < 0.02).  Women with depression also had worse scores  (11.6+7.6 vs. 22.8+5.7, p50.0001).  Despite the high risk for FSD, sexuality had never been previously evaluated in 72.2% of the diabetics  and 77.8% of the controls.  50% of the women with T2DM reported that their sexuality was  significantly affected by the disease. This perception  was correlated with worse FSFI score. Therefore, women feeling that DM had little or no influence on their sexuality had better FSFI scores than those subjects experiencing an important influence (25.5+3.8 vs. 15.2+8.0, p<0.002). | Spanish version of FSFI  BDI |
| Fang SY, Yu SX, Qing LQ, Juan CY, Juan ZH, Ying ZJ. Study on Female Sexual Dysfunction in Type 2 Diabetic Chinese Women. *Biomed Environ Sci*. 2012;25(5):557-561.  In text citation number -29 | China  Funding: Research Fund from Zhejiang Health Department | To investigate the female sexual dysfunction (FSD) in type 2 diabetes patients, by comparing  the sexual function between type 2 diabetic women and non‐diabetic women with Female Sexual  Function Index (FSFI). | Outpatient clinic of three major medical centres of Suzhou and Hangzhou | n = 106 Chinese T2DM women  n =100 Chinese non-diabetic matched by age and background | Case-control | Independent *t*‐test;  Pearson’s chi‐square test; logistic regression | T2DM | Premenopausal/  post-menopausal | **Pre‐menopause group**s  28 women with FSD in non‐diabetic controls (41.2%)  43 with FSD in type 2 diabetic patients (70.5%).  Difference of FSD in pre‐menopause diabetic and  non‐diabetic subgroups were statistically significant  (*P*<0.01).  **Post‐menopause groups**  27 women with FSD in  non‐diabetic controls (84.4%),  41 with FSD in  diabetic patients (91.1%)  The difference was not statistically significant (*P*>0.05):  **Total FSD**  55% FSD women in non‐diabetic controls, and 79.2% FSD women in diabetic patients, with statistically significant difference (*P*<0.01).  **FSFI total score and six aspects**  More fertile women with diabetes have problems in Desire, Arousal, Satisfaction and the total FSFI score, while the difference in post‐menopause women with or without diabetes only exists in the dimension of Orgasm.  Regression analysis indicated age (regression coefficient=1.135, *P*=0.000) and diabetes (regression coefficient=3.480, *P*=0.002) were independent risk factors of FSD.  Haemoglobin A1c did not enter the regression equation.  BMI (regression coefficient=0.842, *P*=0.031) entered the regression equation only in the diabetic group, implying a possible relation between obesity and FSD in diabetes women. | FSFI |
| Mackay L. A grounded theory approach to explore how women with Type 1 diabetes manage their diabetes during the menopausal transition. *Grounded theory approach to explore how women with Type 1 diabetes manage their diabetes during the menopausal transition*. Published online January 2012:N.PAG p-N.PAG p.  In text citation number - 6 | UK  Funding: not reported | To explore the experiences of women with T1DM during the menopause transition and to explore any effect on DM management | Scottish Diabetes clinic | n = 10  White women aged 49-55 (median 51.5) – recruited via hospital data base | Qualitative interviews | Constant comparative Analysis – Grounded Theory | T1DM |  | • **Lack of Information:** A central problem identified was the "blank wall," which refers to the absence of information regarding menopause and its impact on type 1 diabetes. Women felt a lack of evidence-based information from healthcare professionals (HCPs) and desired more guidance on what to expect.  • **Glycaemic Control:** The menopausal transition led to unpredictable blood glucose levels, often described as a "juggling game". Women experienced erratic blood glucose levels, hyperglycaemia, and found it challenging to manage their diabetes effectively.  • **Anxiety and Fear:** Women reported significant anxiety and fear related to the "unknown" aspects of menopause, the inability to control their diabetes, and the potential for acute complications, particularly hypoglycaemia. Some confused menopausal symptoms, such as hot sweats, with hypoglycaemia.  • **Symptoms and Management:** Participants experienced typical menopausal symptoms like hot flushes, night sweats, and mood swings, which sometimes masked crucial signs of hypoglycaemia. Despite their best efforts, women found it difficult to manage their diabetes due to these changes. Some women also expressed concerns about the impact of diabetes on menopause and hormone replacement therapy (HRT).  • **Healthcare Professional Interactions:** Many women felt that medical staff minimized the hormonal effect on diabetes and ignored the impact of menopause. They desired a more holistic approach to care and felt dismissed when raising concerns about menopause. There was a perceived lack of expert advice regarding HRT, with some believing it was contraindicated due to their diabetes.  • **Ageing and Mortality:** The menopausal transition brought feelings of "I'm old," signifying a negative physical change associated with ageing and an increased awareness of mortality. Women expressed fear regarding the long-term complications of diabetes, which became more apparent during this period.  • **Desire for Support:** Women expressed a strong desire for peer support and found it beneficial to hear how other women coped with similar experiences. They also felt it would be easier to discuss menopause with a female doctor. | N/A |
| Hasan SS, Ahmadi K, Santigo R, Ahmed SI. The validity of the menopause-specific quality of life questionnaire in women with type 2 diabetes. *Climacteric*. 2014;17(4):456-464. doi:10.3109/13697137.2013.864269  In text citation number - 31 | Malaysia  Funding: none | To examine the validity and reliability of the Menopause-specific Quality of Life (MENQOL) questionnaire in a sample of women with diabetes in Malaysia, with the secondary aim of determining whether MENQOL domain scores were associated with depression and diabetes | Outpatient clinics  at PutraJaya Hospital in PutraJaya, Tuanku Jaa ’ far Hospital  and Seremban Health Clinic, both in Negeri Sembilan, one of  the states of Peninsular Malaysia | Total n= 337 post-menopausal women (35-75 yrs)  T2DM n= 241  Control n= 96 | Case-Control | Principle Component analysis (Bartletts test, KMO, Cronbach’s a), independent *t*-test  Logistic regression analysis determined the degree to which depression symptoms and diabetes were associated with quality of life on the MENQOL domains. | T2DM | Post-menopausal | Validity of the MENQOL psychosocial and physical  domains were demonstrated through strong relationships with  the equivalent SF-12 mental and physical component scales.  Results indicated that better SF-12 MCS and PCS scores  were significantly related to lower psychosocial and physical symptoms of the MENQOL.  Diabetes was not a significant covariate and did not change the association between SF-12 component scales and MENQOL physical and psychosocial domains, suggesting that presence or absence of diabetes does not change the quality-of-life measures on the MENQOL instrument.  Observations: symptoms associated with both diabetes and the postmenopausal phase had significantly higher mean scores in diabetes subjects compared to control subjects. The psychosocial  domain was significantly associated with depression,  suggesting that scores on this domain of the MENQOL may  be indicators of depression symptoms | MENQOL – Menopause-Specific Quality of Life questionnaire  Centre for Epidemiologic Studies Depression Scale (CES-D)  Outcomes Survey Short Form (SF-12) |
| Rouen PA, Krein SL, Reame NE. Postmenopausal symptoms in female veterans with type 2 diabetes: Glucose control and symptom severity. *Journal of Women’s Health*. 2015;24(6):496-505. doi:10.1089/jwh.2014.4863  In text citation number - 32 | USA  Funding: Department of Veterans Affairs, Veterans Health Administration, Health Services Research and Development Service Quality Enhancement Research Initiative Program RRP 07-354; the University of Detroit Mercy, Dean's Intramural Funding Award; the University of Michigan School of Nursing, Office of Doctoral and Post-Doctoral studies and Sigma Theta Tau, Lambda Zeta chapter. | To describe the postmenopausal symptom experience in U.S. women with type 2 diabetes and to examine the association between glucose control and symptom severity. | US Veterans - medical record system used by the Veterans Health Administration (VHA) | Ages 45–60 years  Participants were on average 55 years of age, obese, and 11.3 years post-menopause. The majority of participants were white (58%), with a range of other ethnic/minority groups represented  Total sample n = 327  No diabetes n = 90,  T2DM n = 237  HbA1c ≤ 7%, n = 135  HbA1c > 7%, n = 102 | Cross-sectional | Chi-Squared, t-tests, ANOVA, post hoc Bonferroni correction, associations between glucose control and menopause symptoms assessed using multiple regression | T2DM | Post-menopausal  Among women with natural cessation of menses, the average age of menopause was 49.2 years, which did not differ by diabetes status. More than half of the respondents (55.1%; n = 179) reported an induced menopause for surgical or other reasons (trauma, drug exposure) at an early age (mean 39.1 years). Hysterectomy features were similar across all three groups | **Menopausal Symptom Severity**  Headaches and anxiety were more prevalent in those with elevated HbA1c values.  Across all groups symptom severity scores were highest for muscle and joint aches followed by hot flashes, and trouble sleeping; head- aches received the lowest severity scores  Women with worse glucose control demonstrated more severe anxiety and sleep disturbances than those with better glucose control, more severe mood swings than women without diabetes, and more intense headaches compared with both groups  Total MSL severity scores were higher in diabetic women with worse glucose control compared with the other two groups  **Factors associated with symptom severity**  Among women with diabetes, worse glucose control (b = 0.15; p = 0.03), smoking, and a diagnosis of altered mood demonstrated a positive association with perceived menopause symptom severity after adjustment for other covariates. | Menopausal Symptom List (MSL)  12-item Short Form Health Survey  Depressive symptoms: short form of the Centre for Epidemiologic Studies Depression Scale-10, Perceived anxiety: Generalized Anxiety Disorder scale  Perceived Stress Scale-10 measured the respondent’s appraisal of life stressors. |
| Hasan SS, Thiruchelvam K, Ahmed SI, Clavarino AM, Mamun AA, Kairuz T. Psychological health and menopause-specific quality of life of Malaysian women with type 2 diabetes. *Asian Journal of Psychiatry*. 2016;23:56-63. doi:10.1016/j.ajp.2016.07.005  In text citation number - 30 | Malaysia  Funding: not reported | To determine the association between  symptoms of depression and anxiety and type 2 diabetes, and  MENQOL domains and type 2 diabetes using case-control data; and to compare the symptomatology of menopause in post-menopausal women with and without type 2 diabetes | Three Medication Therapy Adherence Clinics (MTACs), two  hospital-based (Hospital Putrajaya and Hospital Tuanku Jaa’far Seremban) and one Health Clinic (Health Clinic, Seremban) in West Malaysia. Sampling was extended to the  Seremban region to include patients of Chinese and Indian  ethnicity | 320 T2DM women over 35: Malay (n=98), Indian (n=117, Chinese (n=99), other (n=6)  320 Non-DM women over 35: Malay (n=67), Indian (n=83, Chinese (n=160), other (n=10) | Comparative | Chi-Square, Fishers, *t-*test, logistic and multiple regression | T2DM | PRE/POST meno (excluded peri due to small numbers)  378 women (with diabetes = 258, control = 120) had attained menopausal status, while 262 women (with diabetes = 62, control = 200) had not attained menopausal status. | Women with diabetes had slightly higher depressive (11.8%  versus 8.4%) and anxiety symptoms (8.4% versus 6.6%) compared to  women without diabetes. Postmenopausal women in the diabetes  group and premenopausal women in the control group had slightly  higher proportions of depressive and anxiety symptoms compared to their counterparts.  In the diabetes group, most of the depressed and anxious  participants were aged 45–54 years (27.0% and 27.5% respectively).  Anxiety was more commonly reported by  Chinese women (30%), while depression was more frequently  found among Indians (19%). In the non-diabetes group, most of the  depressed and anxious participants were older, of Indian origin,  In the multivariate logistic regression model, the association  between depressive symptoms and diabetes was not statistically  significant, both in postmenopausal (OR 0.39, 95% CI: 0.13–1.17) as  well as in premenopausal women (OR 0.27, 95% CI: 0.10–1.33)  No significant association between anxiety symptoms and diabetes, both in postmenopausal (OR 0.46, 95% CI: 0.12–1.71) and premenopausal women (OR 0.91, 95% CI: 0.10–8.62).  **MENQOL findings (Post-menopause group case = 258, control = 120):**  The odds that a postmenopausal woman with diabetes was  depressed on the DSSI scale increased 1.13 times when the  MENQOL score on the vasomotor domain increased by one unit (95% CI: 1.04–1.22), the odds ratio increased 1.14 times when the MENQOL score on the psychosocial domain increased by one unit (95% CI: 1.07–1.21), and the odds ratio increased 1.08 times when the MENQOL score on the physical domain increased by one unit (95% CI: 1.04–1.11)  Most common menopausal symptom / was aches of muscles and joints (>70%) pattern of occurrence of specific menopausal symptoms in postmenopausal women, women with diabetes reported significantly higher percentages compared to women without diabetes for the following symptoms: nights sweats (32.9% versus 21.7%, p = 0.025), decrease in physical strength (72.1% versus 56.7%, p = 0.003), decrease in stamina (73.3% versus 53.3%, p = 0.001), lack of energy (73.6% versus 54.2%, p = 0.001), and frequent urination (51.9% versus 27.5%, p = 0.001).  Women with diabetes had insignificantly higher scores for  vasomotor (6.17 versus 5.75, p = 0.357) and physical (37.01 versus  36.75, p = 0.884) domains compared to women without diabetes.  summary  About 40% of those with type 2 diabetes had experienced menopause compared to only 18% of those without diabetes, suggesting that an earlier onset of diabetes results in an earlier age of menopause.  Night sweats, decreased physical strength, decreased stamina, lack of energy, and frequent urination were the predominant menopausal symptoms reported by our participants with diabetes.  women with diabetes reported higher percentages of  depression (11.8% versus 8.4%) and anxiety symptoms (8.4% versus 6.6%) compared to women without diabetes, the differences were statistically insignificant. Similarly, anxiety (31.8% versus 33.3%) and depression symptoms (20.9% versus 20.8%) as measured by the psychosocial domain of MENQOL were also similar for both groups, suggesting that attainment of menopausal status did not increase or decrease the psychological burden in participating women. (NB - From the clinical perspective, the results  indicate that symptoms of depression and anxiety are more prevalent among women with diabetes than women without  diabetes.  Depression was more prevalent among women of Indian ethnicity (19%), while anxiety was more prevalent among the Chinese ethnicity (30%) | MENQOL |
| Parsa P, Ahmadinia Tabesh R, Soltani F, Karami M, Khorami N. Effects of Group Counseling on Self-care Behaviors in Menopausal Women with Diabetes. *Journal of menopausal medicine*. 2017;23(2):108-116. doi:10.6118/jmm.2017.23.2.108  In text citation number - 33 | Iran  Funding: not reported | To investigate the effect of counselling on self-care behaviours in menopause diabetes women in 2016 in Hamadan, Iran | Menopausal women with type II diabetes  who referred to Diabetes Research Centre of Hamadan  city | Total sample: N = 80  Control: n= 40  Intervention: n=40  The mean ± SD age of postmenopausal women in experimental group was 55.0 ± 5.3 and in control group was  55.0 ± 4.6 years (P > 0.05)  32 participants had a history of diabetes for 1 to 5 years and only 9 patients had more than 15 years history of diabetes.  randomly selected and allocated into two groups (the experimental and  the control groups) | Quasi-experimental  Self-care intervention: four  one-hour sessions of group counselling | Independent and paired *t-*tests and ANOVA | T2DM | Menopause | the mean ± SD score of self-care in the experimental group was at weak level that increased to moderate level after the intervention (from 49.6 ± 12.2 to 66.6 ± 9.3; P < 0.001).  Self-care score in the control group  increased from 57.6 ± 11.5 to 62.0 ± 11.6 (P < 0.05)  Comparing the two groups on self-care scores showed  significant differences after the intervention (P < 0.001).  Intervention group scores of diet, exercise, blood sugar control and foot care increased significantly (P < 0.001), except  in the domain of medication that not changed significantly  (P = 0.148). No significant changes were seen in the control group.  Average of fasting blood sugar (FBS) in the experimental group significantly decreased from 148.8 ± 62.4 to 128.9 ± 46.3 (P < 0.001). No significant change in FBS was observed in the control group (P = 0.861). Comparison of two groups after three months showed that the average amount of HbA1c decreased in experimental group, however no significant difference was seen between two groups in term of HbA1c three months after intervention (P = 0.052). A significant difference was observed between the two groups in term of FBS after three months of intervention (P < 0.01). | Summary of Diabetes Self-  Care Activities (SDSCA). The self-care questionnaire developed by Toobert and Glasgow (1994) and included 21 questions about individual caring activities. |
| Inuy AA, Menezes AM, Filho FF, M da SCA, Pardini D. Evaluation of sexual function of postmenopausal Brazilian women. *International Journal of Women’s Health and Reproduction Sciences*. 2018;6(2):140-143. doi:10.15296/ijwhr.2018.23  In text citation number - 11 | Brazil  Funding: None | Analyse the impact of diabetes on the sexual function of postmenopausal Brazilian women. | The National Association of Diabetes Care and Menopause Outpatient Clinic at Federal University of São Paulo | 184 women with a postmenopausal status were subdivided into groups with T2DM (n = 100) and without (n = 84) (diabetes group and control group, respectively).  Mean age – 56 + 5 (diabetic group) 55+ 4 (control)  Diabetes duration 11 + 6 yrs  Diabetic group criteria: a diagnosis of T2D, absence of severe chronic diabetic complications that the participant related as a limiting factor for sexual practice, HbA1c levels < 8.0%, and absence of insulin therapy. | Cross sectional retrospective study (2013-2015) |  | T2DM | postmenopause | SD prevalence was significantly greater in the group with T2DM (85%) versus the group comprising control participants (69%) (P=0.001; relative risk [RR] 1.28, 95% confidence interval 1.09–1.51).The mean overall FSFI score was lower in the T2D group (13.48 ± 10) versus the control group (18.14 ± 11; P=0.004).  Specific items in the questionnaire, significant differences were found in the diabetic group for decreased arousal (P=0.004), desire (P=0.001), lubrication (P=0.015), orgasm (P=0.019), satisfaction (P=0.002), and pain (P=0.002). SD was associated with T2D duration in the diabetes group and with age (P=0.001) and menopause duration (P=0.001) in both the groups. | The Female Sexual Function Index (FSFI) |
| Slopien R, Wender-Ozegowska E, Rogowicz-Frontczak A, et al. Menopause and diabetes: EMAS clinical guide. *Maturitas*. 2018;117:6-10. doi:10.1016/j.maturitas.2018.08.009  In text citation number - 9 | Greece  Funding: none | To summarise the evidence on 1) the effect of menopause on metabolic parameters and the risk of T2DM,  2) the effect of T2DM on age at menopause, 3) the effect of MHT on the risk of T2DM, and 4) the management of  postmenopausal women with T2DM | N/A | N/A | Literature review and consensus of expert opinion | Narrative Synthesis | T2DM | Peri/Menopause | MHT improves glycaemia, insulin resistance and other components of the metabolic syndrome. Despite this, the frequency of MHT use among women with T2DM is about 50% lower than in the general population.  An individualised approach in treating menopausal symptoms  should be considered. Women with T2DM may be excellent candidates for MHT, after careful evaluation of their CVD risk.  Adverse metabolic consequences of the menopause can  be countered by lifestyle changes, such as optimal diet, increase in physical activity, cessation of smoking and decrease in alcohol consumption. Lifestyle intervention should be the cornerstone of management in women with T2DM entering the menopause. |  |
| Szadowska-Szlachetka ZC, Stasiak E, Leziak A, et al. Intensity of menopausal symptoms and quality of life in climacteric women. *Przeglad menopauzalny = Menopause review*. 2019;18(4):217-221. doi:10.5114/pm.2019.93113  In text citation number -34 | Poland  Funding: not reported | To evaluate the quality of life of women (aged 48-55 years) in the physical, mental and social spheres, to determine the intensity of menopause symptoms and to ascertain whether the above indicators are influenced by sociodemographic factors. |  | n= 161 women from the Lublin region who do not use hormonal replacement therapy. 83 of them (52%) were 48-51 years old, and 78 (48%) were 52-55 years old. Women whose last menstruation had occurred within 1-2 years before constituted the most numerous group. A total of 37.9% of women had last menstruated 2-5 years before and those whose last menstruation period had been more than five years before comprised the largest group (15.5%). NB: only n= 10 (12.8%) of the sample had diabetes | Cross-sectional |  |  | Not specified | Respondents living with diabetes rated their quality of life significantly lower compared to those without DM including their performance  in the following domains of life: physical health  (p = 0.000924), environment (p = 0.014370) and general  satisfaction with health status (p = 0.020624).  Diabetes had a statistically significant effect on menopausal symptoms but the individual symptoms were not reported. | WHOQOL-BREF  Kupperman index - measures the severity of menopause symptoms |
| Yang YL, Chee W, E.-O. I. Type 2 Diabetes and Musculoskeletal Symptoms Among Midlife Women. *The Diabetes educator*. 2019;45(5):520-528. doi:10.1177/0145721719872558  In text citation number - 35 | USA  Funding: Secondary analysis of the quantitative data from two larger studies that were funded by the National Institute of Health | To describe the association between type 2 diabetes and musculoskeletal symptoms among midlife women from four major racial/ethnic groups in the U.S. |  | n= 164 Hispanic, Non-Hispanic, White, African American or Asian midlife women (62 with T2DM and 102 without T2DM) | Secondary analysis of data retrieved from two large cross-sectional nationally representative internet surveys on midlife women of four major racial/ethnic groups in the U.S. | Independent t-test and Mann–Whitney U test were used to assess differences in total scores of the number and severity of musculoskeletal symptoms between groups (T2DM status, menopausal status, and race/ethnicity)  Hierarchical multiple regression analysis was performed to examine the association between T2DM and musculoskeletal symptoms while controlling for confounding factors including background characteristics, health status, and menopausal status. | T2DM | Pre-menopausal: 19.5%    Early or late peri-menopausal: 34.8%  Post-menopausal -45.7%,  Premenopausal: menstruation in the past 3 months with no  change in regularity, premenopausal.  Early peri-menopausal: menstrual bleeding in the past 3 months but had increasing changes in cycle length over the  past year  Late perimenopausal:  menstruation in the past 12 months but not in the past 3 months  Postmenopausal: no menstruation in the past year | The mean number of symptoms was significantly higher in women with T2DM (3.69 ± 2.57) than in those without T2DM (2.07 ± 2.09, t = 4.20, p < .001), as was the mean severity score (12.19 ± 9.98 and 6.04 ± 6.98, respectively; t = 4.26, p < .001).  Effects of T2DM Status on the Number and Severity of Musculoskeletal Symptoms According to Menopausal Status:  Significant regardless of menopausal status - women with versus without T2DM (premenopausal: U = 57.00, p = .024; perimenopausal: U = 242.50, p = .009; and postmenopausal: U = 410.00, p = .008) (overall p value < .05).  The mean severity score for musculoskeletal symptoms was also significantly higher in women with versus without T2DM in all three subgroups (premenopausal: U = 55.00, p = .021; perimenopausal: U = 261.50, p = .023; and postmenopausal: U = 397.00, p = .005) (overall p value < .05).  Severity according to race  The mean severity score for musculoskeletal symptoms was significantly higher in Non-Hispanic (N-H) white women with T2DM than in those without T2DM (U = 123.00, p = .042) (p < .05) (Table 4). T2DM status had no effect on the number or severity of musculoskeletal symptoms in the Hispanic, N-H Asian, or N-H African-American groups (all p values > .05).  When controlled for background characteristics and health status, a significant and positive association was found between the number of musculoskeletal symptoms and T2DM (β = .245, p < .001) |  |
| Zimny M, Starczewska M, Szkup M, Karakiewicz-Krawczyk K, Grochans E, Sipak-Szmigiel O. Analysis of the impact of type 2 diabetes on the psychosocial functioning and quality of life of perimenopausal women. *International Journal of Environmental Research and Public Health*. 2020;17(12):1-17. doi:10.3390/ijerph17124349  In text citation number - 36 | Poland  Funding: Pomeranian Medical University in Szczecin, Poland | To identify the link between body composition, the severity of depressive and climacteric symptoms and the quality of life in healthy women and those suffering from type 2 diabetes in the perimenopausal period. | Women from West Pomeranian Voivodeship. | N = 172 women in the perimenopausal period aged age 45 - 65.  Study group: included 68 women with diagnosed type 2 diabetes, and 104 healthy participants constituted  the control group.  Study group average age was 58.7, while the average age in the control group was 52.3.  Inclusion: type 2 diabetes; no thyroid, mental or cancerous  diseases at the time of the study or in the patient’s history as well as no history of bariatric surgery | Diagnostic poll method |  | T2DM | Perimenopause | Combined analysis of all surveyed women showed that the majority did not present symptoms of depression (77.9%). However, 15.1% showed mild, 4.7% presented with average  and 2.3% with severe symptoms of depression. No statistically significant differences were observed  between the study and control group as far as depressiveness was concerned (p > 0.05).  Study group, the quality of life was lower in the following  domains: physical functioning (P.F.), role limitations due to physical problems (R.P.), bodily pain (B.P.), general health perception (G.H.) and physical functioning, or physical component summary (P.C.S.).  The greatest differences between the study and control group were observed within R.P. and R.E. (role  limitation due to emotional problems). | The Blatt–Kupperman Index,  The Beck Depression Inventory  The Short-Form-36 Health Survey (SF-36) |
| Anderson D, Sturt J, McDonald N, et al. International feasibility study for the Women’s Wellness with Type 2 Diabetes Programme (WWDP): An eHealth enabled 12-week intervention programme for midlife women with type 2 diabetes. *Diabetes Research and Clinical Practice*. 2021;171:108541-108541. doi:10.1016/j.diabres.2020.108541  In text citation number -37 | AUS/  UK  Funding: Diabetes Queensland &  Internal funding at King’s College London. | To examine feasibility of participant recruitment and retention  rates for the Women’s Wellness with Type 2 Diabetes program (WWDP), and to assess initial efficacy of the programme in improving wellbeing outcomes | Diabetic women enrolled in a wellness programme based on SCT to increase self-efficacy to promote healthy behaviours | n= 56 Women aged 45–65 years with T2DM recruited from Australia and the UK through advertisements using regular and social media, diabetes charities, women’s membership organisations and employers | Pre/post  Intervention | Chi-square, McNemar, paired t-test, and Wilcoxon signed-ranks tests. | T2DM | Not specified | *Menopausal symptoms.* Significant reductions in total menopausal symptoms (*z* = -3.58, *p* < .001) from baseline.  Significant reductions were found in all subscales, including the psychological (total: *z* = -2.95, *p* < .01; anxiety: *z* = -2.39, *p* < .05; depression: *z* = -2.69, *p* < .01), somatic (*t*(53) = 2.52, *p* < .05), [vasomotor](https://www.sciencedirect.com/topics/medicine-and-dentistry/vasomotor) (*z* = -3.77, *p* < .001), and sexual dysfunction (*t*(53) = 2.08, *p* < .05) symptoms.  When examined by country, reductions remained significant for total menopausal (AU: *t*(26) = 2.25, *p* < .05; UK: *z* = -2.63, *p* < .01), total psychological (AU: *z* = -2.18, *p* < .05; UK: *z* = -2.04, *p* < .05) and vasomotor (AU: *z* = -2.37, *p* < .05; UK: *z* = -2.93, *p* < .01) symptoms. Reductions also remained significant for the UK sample for depression (*z* = -2.35, *p* < .05) and sexual dysfunction (*t*(26) = 2.10, *p* < .05) symptoms.  Diabetes distress. Significant reductions in total DD (z = -4.88,  p < .001) were observed from baseline. Significant reductions  were also noted in regimen-related DD (z = 4.59, p < .001),  emotional burden (z = -4.28, p < .001) and physician-related  DD (z = 2.05, p < .05).  Significant improvements in self-reported general  health (t(53) = 4.24, p < .001), vitality (t(53) = 3.27, p < .01), phys-  ical function (z = 2.76, p < .01), reported health transition  (z = 4.37, p < .001), social function (z = 2.20, p < .05) mental  health (z = 2.91, p < .01) and overall mental component score  (z = 2.67, p < .01) from baseline. Other domains did not differ  significantly over time (p = .08-0.38). | Greene Climacteric Scale  Diabetes Distress scale  SF-36: health related QoL |
| Escobar Florez OE, Aquilera G, De la Roca-Chiapas JM, Macías Cervantes MH, Garay-Sevilla ME. The Relationship Between Psychosocial Factors and Adherence to Treatment in Men, Premenopausal and Menopausal Women with Type 2 Diabetes Mellitus. *Psychol Res Behav Manag*. 2021;14:1993-2000. Published 2021 Dec 11. doi:10.2147/PRBM.S342155  In text citation number - 38 | Mexico  Funding:  University of Guanajuato. | To describe the psychosocial factors that may affect adherence to treatment among men and  premenopausal and menopausal women with T2DM. |  | 96 patients with T2DM, who were divided  into three groups: 1) men (n=32); 2) premenopausal women (n=32); and 3) menopausal  women (n=32).  Inclusion/exclusion: age between 40–64 years; more than 5 years since the diagnosis of T2DM; more than 6 years of education; no history of vascular disease, stroke, psychiatric or neurological disease, visual or hearing impairment, or major depressive disorder; no consumption of alcohol or tobacco; no beta-blocker, neuroleptic, vitamin E, or hormone replacement therapy. | Cross-sectional | Differences between the groups of men and premenopausal and menopausal women were assessed using the ANOVA test for parametric variables and the Kruskal–Wallis test for non-parametric variables along with the Tukey post hoc test. Spearman correlation was used to assess adherence to medication and diet with psychosocial factors. | T2DM | Pre-menopausal and menopausal | Significant difference in social support between men and menopausal women (p <0.02) and in depressive symptoms between men and menopausal women (p <0.015). In general, men had greater social support than menopausal women, and menopausal women had more depressive symptoms than men. A marginal difference was observed for disease denial, which was greater among men.  Adherence to medication had a negative correlation with depressive symptoms among men (p <0.001) and menopausal women (p <0.021); no association was found for the other psychosocial factors.  Older menopausal women perceive less social support, which can predispose them to more menopausal and depressive symptoms.28 Premenopausal and menopausal women experience phases of large hormonal fluctuations in estradiol and progesterone levels.  Social support did not show a significant relationship with adherence to treatment in any of the groups.  Menopausal women showed greater depressive symptoms and less social support than men. |  |
| Tirupathi. PK, Dhanapal. CK, Muvvala. S, Pratap. D, Bejugam. S, Raghavendra. K (2021) Assessment of the menopausal symptoms and their effect on the quality of life in diabetic postmenopausal osteoporosis women. J. Global Trends Pharm Sci, 2021; 12 (2): 9387 – 9394  In text citation number - 39 | India  Funding: not reported | To assess the Quality of Life of the Postmenopausal Woman with diabetes. To estimate the prevalence of osteoporosis in Diabetic postmenopausal women, categorized into three groups: Normal, Osteopenia and Osteoporotic groups |  | N = 520 Diabetic Postmenopausal women - age group more than 45 Years of all weight groups and Postmenopausal women using either insulin or oral hypoglycemic agents.  114 patients were of normal bone density, whereas 317 patients were of Osteopenia condition and 89 patients were of Osteoporosis condition: based on Bone Mineral Density assessment |  |  | Not specified | Post-menopausal | hot flushes (28.6%), experiencing poor memory  (62.5%), being dissatisfied with their personal life (71.3%), Low backache  (56.6%), and change in sexual desire (18.4%).  The highest mean QoL score was identified for sexual domain followed by psychosocial features. | MENQOL |
| Yoshida Y. Early Menopause and Cardiovascular Disease Risk in Women With or Without Type 2 Diabetes: A Pooled Analysis of 9,374 Postmenopausal Women. 2021;44.  In text citation number - 40 | USA  Funding: Supported by a grant (NIH K12HD043451) from the Eunice Kennedy Shriver National Institute of Child Health & Human Development of the Building Interdisciplinary Research Careers in Women's Health (BIRCWH) Scholar. FMJ was supported by NIH grants (DK107444 and DK074970), a U.S. Department of Veterans Affairs Merit Award (BX003725) and the Tulane Centre of Excellence in Sex-Based Biology & Medicine. AHA was supported by NIH grants (R01DK104730, R01DK107566 and P20GM109036) and the Tulane University Translational Science Institute. | To examine CVD risk in women with early compared with normal-age menopause, with and without T2DM overall, and by race/ethnicity. | Pooled from three major U.S. prospective studies of CVD: the Atherosclerosis Risk in Communities (ARIC) study, the Multi-Ethnic Study of Atherosclerosis (MESA), and the Jackson Heart Study (JHS). | N = 9374 postmenopausal women - (T2DM, n= 1,237;  N = non-T2DM, 8,137). Mean age of 58. Self-reported race among the postmenopausal women was 58%, 32%, and 10% for White, Black, and other, respectively. In the T2DM group, there was a higher pro- portion of Black than White women or women of other racial groups (52% vs. 35% and 13%, respectively). In the non- T2DM group, there were more White than Black women or women of other racial groups (61% vs. 29% and 10%, respectively). | Observational -Cohort study | x² and t tests to compare  the differences of characteristics between  postmenopausal women with and with-  out T2DM.  Poisson  Regression compare  the difference in incidence rate between  menopause at <45 and $45 years of  age.  association between early meno-  pause and CVD outcomes was examined  in Cox proportional hazards models. The  model was first adjusted for age and  race/ethnicity and then further adjusted  for CVD risk factors, including smoking  status, alcohol drinking status, blood pres-  sure, total cholesterol, BMI, ever used  MHT, ever pregnant, and cohort indicator.  Interaction terms between early meno-  pause and T2DM were added to models  to test the potential modifying effect of  T2DM on early menopause and CVD out-  comes. | T2DM | Postmenopausal | Compared with women  without T2DM, individuals with T2DM  were more likely to have experienced early  menopause (41% vs. 36%), have had  surgical menopause (42% vs. 37%)  Black women reported meno-  pause onset at a younger age and had  the highest proportion of surgical meno-  pause among the three racial groups in  the cohort. White women were more  likely to use MHT than the Black and other racial groups  Women with T2DM were less likely to use MHT at baseline than  women without T2DM  In women experiencing early menopause T2DM was a factor  median follow-up of 15 years  for 9,374 postmenopausal women, 1,068  CHD, 659 stroke, 1,412 HF, and 1,567  ASCVD events were observed. Women  with early menopause experienced signif-  icantly higher crude CHD, ASCVD, and HF  event rates (incidence per 1,000 person-  years) than those who had menopause at  normal age. Women with T2DM  who reported early menopause showed  significantly higher incident rates across  all outcomes than those who reported  menopause at normal age with or with-  out T2DM Age- and race-adjusted associations  between early menopause and all out-  comes were statistically significant (each  P < 0.05)  T2DM significantly modified the  relationship between early menopause  and CHD, stroke, and ASCVD (P for inter-  action ≤0.05).  association between  early menopause and the incidence of  CVD by T2DM. The hazard of CVD associated with early menopause was more  evident in women with T2DM than  those without, particularly among Black  participants. |  |
| Lambrinoudaki I, Paschou SA, Armeni E, Goulis DG. The interplay between diabetes mellitus and menopause: clinical implications. *Nature reviewsEndocrinology*. 2022;18(10):608-622. doi:10.1038/s41574-022-00708-0  In text citation number - 41 | Greece  Funding: Not reported | aims to critically appraise the literature on the association  between diabetes mellitus and menopause, and to answer  five key questions. Does diabetes mellitus affect ovarian  ageing or the onset of menopause? Does the risk of  developing diabetes mellitus increase after menopause,  independently of age? Is diabetes mellitus associated  with the severity of menopausal symptoms and do menopause and diabetes mellitus interact as cardiovascular  disease (CVD) and osteoporosis risk factors beyond their independent effect? Does the management of menopause  with menopausal hormone therapy (MHT) differ in women with and without DM.  Finally, does the management of diabetes mellitus differ  in postmenopausal compared with its management in  premenopausal women and men? |  |  | Review |  |  |  | MHT might decrease the risk of incident T2DM and  improve glycaemic control in women with pre- existing DM.  Postmenopausal women with diabetes mellitus have a higher risk of CVD associated with diabetes mellitus than age- matched men with diabetes mellitus, and higher fracture risk than women without diabetes mellitus, independently of  Bone mineral density.  In women with diabetes mellitus and  indications to receive MHT, such as bothersome menopausal  symptoms, vulvovaginal atrophy or a diagnosis of  premature ovarian insufficiency, MHT should be considered  after CVD risk stratification. The transdermal  route is preferred in women with long- standing diabetes  mellitus, obesity or other cardiovascular risk factors. |  |
| Forde R, King L, Cooper A, Winkley K, Coates V. Type 1 diabetes and menopause, what women would like to know: a public and patient involvement exercise. *idn*. 2024;17. doi:10.57177/idn.v17.327  In text citation number - 42 | UK  Funding: none | To gain an understanding about what is important to women with Type 1 diabetes in relation to menopause and perimenopause | Online diabetes community with majority UK | 184 responses with representation from each region across the United Kingdom (UK) and over 20% (*n* = 39) from outside of the UK  Aged between 40 and 59 years (*n* = 167, 90.8%) with 6.5% (*n* = 12) aged 60 years or older and the remainder less than 39 years (*n* = 5, 2.7%). | PPI exercise involving an online open discussion | Categorical data analysed descriptively through SPSS.  Open text question was analysed thematically using Microsoft Excel for data management. Coding frequency based on analysis of initial sample. Codes were  initially generated on a sample of the responses by three  researchers individually and then discussed until consen-sus reached. The remainder of the dataset was then coded  accordingly. | T1DM | Most women considered themselves to be either peri-menopausal (*n* = 79, 42.9%) or menopausal (*n* = 60, 32.6%); however, 10% (*n* = 19) of women were unsure how to describe themselves in relation to the menopausal trajectory. | Most women (72.8%) reported that menopause had impacted their diabetes  Only a minority of women (27.2%) indicated that menopause had ever been discussed with them during diabetes consultations.  The primary theme identified was an overall lack of awareness about the impacts of menopause, both in general and specifically concerning its interaction with diabetes.  Women expressed a strong need for information about menopause, including general symptoms (mood swings, hot flashes, night sweats, sleep disturbance, fatigue, brain fog, weight gain, anxiety, depression, genitourinary symptoms, loss of libido) and how menopause affects diabetes.  Women felt that menopause added to the burden of managing diabetes, making self-care more problematic due to unpredictable blood glucose fluctuations and changing insulin requirements  challenge of distinguishing between symptoms of hypoglycaemia and vasomotor symptoms of menopause, which often led to suboptimal diabetes management and increased anxiety.  Participants reported difficulties in communicating with healthcare professionals about menopause, feeling that professionals lacked knowledge and often dismissed their concerns. Women desired timely, appropriate, evidence-based care, and signposting to resources, including peer support.  The management of diabetes and menopause is an unmet need within the UK health service, not adequately covered in national guidance, patient education, or routine clinical practice. |  |

AU: Australia; BMI: Body Mass index; ENS: Empty Nest Syndrome; DHEAS: Sulfoconjugated form of the dehydroepiandrosterone;

DM: Diabetes Mellitus; HRT: Hormone Replacement Therapy;T1DM: Type 1 Diabetes Mellitus; T2DM: Type 2 Diabetes Mellitus; FSH: Follicle Stimulating Hormone; NIIDM: Non-Insulin Dependent Diabetes; SMBG: self-monitoring of blood glucose; SBP: Systolic Blood Pressure; UK: United Kingdom
